# Pathways for glomerular macromolecule filtration: A mathematical model for transport across glomerular filtration surface, mesangium and shear-induced shunts

**DOI:** 10.1371/journal.pcbi.1014503

**Published:** 2026-07-20

**Authors:** Numpong Punyaratabandhu, Thapakorn Pankoh, Yuttana Roongthumskul, Panadda Dechadilok, Pisut Katavetin

**Affiliations:** 1 Department of Physics, Faculty of Science, Chulalongkorn University, Wang Mai, Pathumwan, Bangkok, Thailand; 2 Biophysics for Applications in Cellular and Organ Physiology in Humans Research Group, Faculty of Science, Chulalongkorn University, Wang Mai, Pathumwan, Bangkok, Thailand; 3 Division of Nephrology, Department of Medicine, Faculty of Medicine, Chulalongkorn University, Pathumwan, Bangkok, Thailand; Lehigh University, UNITED STATES OF AMERICA

## Abstract

A mathematical model is developed to investigate the relative contribution of macromolecule transport across the glomerular filtration surface and that across mesangial area to glomerular size-selectivity. Endothelial fenestrae are assumed to be filled with glycosaminoglycans. Glomerular basement membrane (GBM) is a hydrogel containing two types of fibers. Slit diaphragm is a row of parallel cylinders with inter-fiber spacing following a lognormal distribution. Glomerular mesangium is viewed as a Brinkman medium with solute diffusivity and convection rate calculated from hydrodynamic forces exerted on confined spheres. Comparison between calculated sieving coefficients and those of Ficolls from *in vivo* studies demonstrates that inclusion of fluxes across the filtration surface and mesangial area, although capable of explaining small and medium-sized solute sieving, underestimates filtration of macromolecules with radii larger than 5 nm. Based on electron micrographs displaying red blood cells escaping through openings at the junction between the filtration surface and mesangium, the location with maximum shear stress, the present study examines effects of these openings using low-Reynolds-number hydrodynamics. Even though such effects on filtration of small and moderate-sized solutes are negligible, the presence of possibly shear-induced openings amplifies sieving of large macromolecules, yielding calculated sieving coefficients that agree well with those obtained from urinalysis in healthy humans and patients with diabetic nephropathy for the entire range of solute radii. While the glomerular filtration surface is the main pathway for small and moderate-sized solutes, main passages of large macromolecules are likely to be through the shear-induced openings, explaining the “upper limit” of glomerular size-selectivity.

## Author summary

Solute restriction of different components of the glomerular barrier is examined.

Calculated results show that the effect of solute transport across mesangium is small.

Glomerular filtration surface is the main pathway for small/moderate-sized solutes, whereas shear-induced shunts at their junction are likely to be where large solutes escape. Computed sieving coefficients agree with *in vivo* studies for all Ficoll radii.

## Introduction

The first step of renal urine formation, the process responsible for removing metabolic waste and fluid from blood circulation, is an ultrafiltration in glomerular capillaries [1]. In healthy humans, the glomerular filtration surface covers approximately 70% of the glomerular capillary surface area [2]. The glomerular filtration surface consists of a fenestrated endothelial cell layer, a glomerular basement membrane (GBM) and an epithelial cell layer [3]. It is commonly referred to as the glomerular filtration surface and believed to be the main pathway for fluid and solute transport. Its abnormality has been associated with various diseases causing nephrotic syndrome; fluid and solute transport across the filtration surface has been extensively studied [4]. For instance, several mathematical models have been developed with an aim of relating the nanostructure of the filtration surface to glomerular selectivity [5–11].

Among the constructed mathematical models, the model with the simplified geometry of the transport barrier most closely resembling the glomerular filtration surface is the ultrastructural model [12–14] where GBM and a slit diaphragm modeled as a row of parallel cylinders with the mean gap-width of the spacing between fibers being close to values reported by Rodewald and Karnovsky [15] are considered to be the primary restriction layers. Later images of the slit diaphragm obtained by Gagliardini et al. [16] from scanning electron microscopy as well as those obtained by Rice et al. [17] from helium ion electron microscopy, however, indicated that the size of the spacing between fibers of the slit diaphragm is approximately 6 times wider than the value reported by Rodewald and Karnovsky [15]. Punyaratabandhu et al. [18,19] examined glomerular size-selectivity by utilizing the ultrastructural model but with the mean gap-width of the spacing between fibers in the slit diaphragm following the value estimated from the reports of Gagliardini et al. [16] and Rice et al. [17] and including transport through endothelial fenestrae. A comparison between the computed results and Ficoll sieving coefficients, the ratio between the Ficoll concentration in Bowman’s space and that in the lumen, from *in vivo* urinalysis [20,21] demonstrates that the calculated sieving coefficients agree very well with experimental results if the solute radii (*r*_*s*_) are less than or equal to 5 nm. For solutes with *r*_*s*_ > 5 nm, however, the calculation is found to underestimate the solute sieving coefficients when compared to those obtained experimentally [19].

Experimental results also demonstrate that there is an “upper limit” of glomerular size-selectivity; based on urinalysis performed in sheep using Ficolls [22], the increase of *r*_*s*_ from 2 nm to 5 nm leads to an almost two-orders-of-magnitude reduction in the Ficoll sieving coefficients. However, when *r*_*s*_ is increased from 5 nm to 8 nm, the sieving coefficients remain approximately constant. The slower decline of the solute sieving coefficients as a function of *r*_*s*_ when *r*_*s*_ > 5 nm is also seen in experimental results from *in vivo* urinalysis in humans [20]. The origin of the glomerular size-selectivity limit remains to be determined. The objective of the present study is to investigate two possible factors that may cause the solute sieving coefficient of large macromolecules from experimental studies to be larger than that predicted by including solute transport across the filtration surface alone. The first possible factor is the contribution of ultrafiltration across the glomerular mesangium. Functioning as a seal that holds the glomerular capillaries together, the mesangium is a critical component of the glomerulus; renal failures have been associated with factors that affecting mesangium functioning [23–26]. In healthy humans, approximately 30% of the glomerular surface area is the four-layered barrier consisting of the three mentioned cellular layers with the addition of the glomerular mesangium located between the endothelial cell layer and GBM [2]. Hunt et al. [27] has modeled the transport of fluid as well as solutes with the same size as those of albumins and Immunoglobulin A (IgA) in the glomerular mesangium; GBM, mesangium and the epithelial slit were assumed to be the main barriers, whereas the contribution of the endothelial cell layer was assumed to be very small. In the present work, the developed computational model for transport of fluid and solutes across the four-layered barrier is inspired by the model of Hunt et al. [27] but with the flux restriction due to the presence of GAGs in the endothelial fenestrae being also included and the range of *r*_*s*_ being 1.6 – 6 nm. The calculated average sieving coefficients, ⟨θ⟩, are, then, compared to Ficoll sieving coefficients from experiments [20,21].

Another factor that may have an impact upon solute sieving into the primary urine is the possibility of the “pores” or “shunts” at the junction between the filtration surface and the glomerular mesangium. Using transmission electron microscopy, Collar et al. [28] obtained electron micrographs showing escapes of red blood cells (RBCs) through the “void” at the junction between the filtration surface and the four-layered barrier (that included the mesangium) from biopsies of glomeruli of patients with sporadic hematuria and thin GBM. RBC escapes were observed at the same location by Liapis et al. [29] for a patient with minimal-change disease. Although the subjects of both experiments were patients with nephrotic syndromes, the subject employed in the work of Liapis et al. [29] had minimal change nephropathy with GBM being quite close to that of healthy humans; Drumond et al. [30] reported that GBM thickness in healthy humans was 518 nm, whereas in patients with minimal change nephropathy, the GBM thickness was 513 nm. It is also reported that a small amount of RBCs is generally found in urine of healthy humans. Utilizing an applied shear flow in microfluidic devices, Lanfer et al. [31] reported that the applied shear flow greatly influenced the orientations of collagens presented in the microchannels. The increase in the volume flow rate resulted in the reduction of the standard deviation of the collagen orientations. Collagens are presented in GBM and are believed to be the main factor contributing to the maintenance of its structure. In the reported micrographs, RBCs are shown to escape through the voids at the junction between the filtration surface and the mesangium, the location with the highest shear flow. Our hypothesis is that it is possible that this shear flow (caused by the change in the fluid velocity at the junction) may lead to an increased collagen alignment, a decrease in GBM structural integrity and a temporal opening of the shunts at the junction between the filtration surface and the mesangium that may allow RBCs to escape. In the present work, the effect of the possibly shear-induced shunts on glomerular size-selectivity is examined. The shunts are assumed to be cylindrical pores that open and close as a function of time, allowing solute transport into the primary urine. Our mathematical model is inspired by the “lognormal distribution and shunt” model [11] and the “charged fiber matrix and large pore model” [32] but with three differences. The first difference is that, in the present study, the fluid and macromolecule transport through the glomerular filtration surface is simulated by employing the ultrastructural model where the filtration surface is assumed to consist of three different layers. The second difference is that the filtration across the four-layered glomerular mesangial area is also included, whereas the third difference is that, in our calculation, the shunts are assumed to be cylindrical pores that open and close as a function of time due to the fact the escaped red blood cells in urine are found to be deformed or even cut in half [28,29]. Total sieving coefficients computed by including the fluxes across the filtration surface, the mesangial area and the shear-induced shunt are, then, compared to Ficoll sieving coefficients from urinalysis performed in healthy humans and patients with diabetic nephropathy [20,21]. Our model is the first mathematical computation that incorporates all three possible pathways for solute transport including the glomerular filtration surface, the mesangium and the possibly shear-induced shunts that transiently open and close. The geometries of all three pathways are based on obtained microscopic images and the employed parameters are physiological parameters of healthy humans and patients with early-but-overt diabetic nephropathy.

## Results

The objective of the present study is to investigate the possible pathway for glomerular filtration of large macromolecules. A mathematical model is constructed to investigate the possible contribution of the flux across the glomerular mesangial matrix and that of the flux through the shear-induced shunt to glomerular fluid and solute filtration. Results presented in this section begin with the examination of the effect of the solute flux across the glomerular mesangium on glomerular macromolecule transport. This is followed by the effect of solute transport through the shear-induced shunt at the junction between the glomerular mesangium and the glomerular filtration surface on glomerular size-selectivity where, as aforementioned, the total sieving coefficients are compared to those of Ficolls from experiments.

### Effects of transport across the glomerular mesangium on glomerular fluid and solute filtration

**Fig 1:**
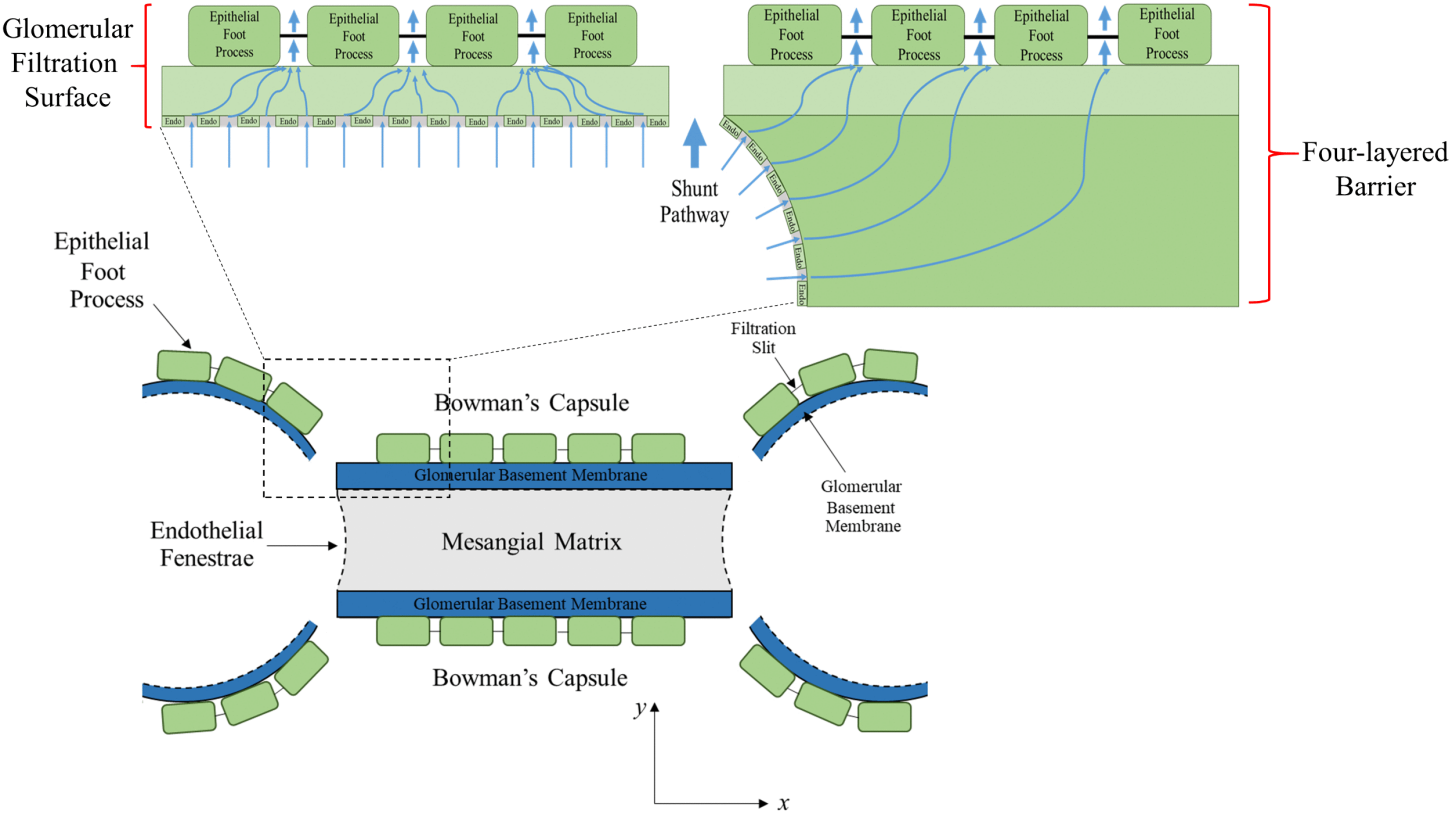
Schematic drawing of the glomerular capillary wall consisting of the glomerular filtration surface, the four-layered barrier with the glomerular mesangium located between the endothelial cell layer and the glomerular basement membrane, and the shear-induced opening at the junction between the two barriers.

In this work, to account for solute transport across the glomerular mesangial matrix, the sieving coefficient calculated by including the solute flux across the filtration surface and that across the mesangial area, ⟨θ⟩, is computed as shown below in Eq. (1).


$$\begin{array}{c} \left\langle \theta \right\rangle =\frac{\left(\theta_{filtration\;surface}S_{fs}\text{\textbackslash{}hspace\{0.17em}\}k_{fs}\text{\textbackslash{}hspace\{0.17em}\;\}\left[\Delta P-\Delta \Pi \right]\right)+\left(\theta_{four-layered}S_{four-layered}\text{\textbackslash{}hspace\{0.17em}\}k_{four-layered}\text{\textbackslash{}hspace\{0.17em}\;\}\left[\Delta P-\Delta \Pi \right]\right)}{\text{SNGFR}} \end{array}$$ (1)

where *θ*_*filtration surface*_ is the sieving coefficient across the filtration surface and *θ*_*four-layered*_ is the sieving coefficient across the four-layered barrier including the glomerular mesangial matrix as shown in Fig 1; the details of their calculations are discussed in Mathematical Method as well as in Sections C and E of S1 Appendix. Bowman’s space is viewed as a space, where, far from the slit diaphragm, the solute concentration at equilibrium is assumed to constant. In absence of the solute concentration gradient, the solute flux is the convective flux; the product of $C_{B}$ (the concentration in Bowman’s space) and the downstream fluid velocity. *k*_*fs*_ and *k*_*four-layered*_ are the hydraulic permeability of the filtration surface and that of the four-layered barrier, respectively; their detailed calculations are given in Mathematical Method and S1 Appendix. *S*_*f*s_ and *S*_*four-layered*_ appearing in Eq. (1) are the surface area of the two barriers, respectively, whereas ΔP is the transcapillary hydraulic pressure difference and ΔΠ is the transcapillary osmotic pressure difference. $S_{fs}\text{\textbackslash{}hspace\{0.17em}\}k_{fs}\text{\textbackslash{}hspace\{0.17em}\;\}\left[\Delta P-\Delta \Pi \right]$ and $S_{four-layered}\text{\textbackslash{}hspace\{0.17em}\}k_{four-layered}\text{\textbackslash{}hspace\{0.17em}\;\}\left[\Delta P-\Delta \Pi \right]$ are the fluid volume flow rate across the filtration surface per glomerulus and that across the glomerular mesangium, respectively, whereas SNGFR is the single nephron glomerular filtration rate. $\left(S_{fs}\text{\textbackslash{}hspace\{0.17em}\}k_{fs}\text{\textbackslash{}hspace\{0.17em}\;\}\left[\Delta P-\Delta \Pi \right]/\text{SNGFR}\right)$ is, therefore, the fraction of the fluid volume flow rate transported across the filtration surface per glomerulus, whereas $\left(S_{four-layered}\text{\textbackslash{}hspace\{0.17em}\}k_{four-layered}\text{\textbackslash{}hspace\{0.17em}\;\}\left[\Delta P-\Delta \Pi \right]/\text{SNGFR}\right)$ is that across the four-layered barrier.

As shown in Fig 1, during macromolecules sieving through the filtration surface, the solutes are filtered through the endothelial fenestrae, across GBM through the slit diaphragm into Bowman’s space. Under the conditions discussed in Mathematical Method, *θ*_*filtration surface*_ is estimated as the product of the solute sieving coefficient across each layer as shown in Eq. (2).


$$\begin{array}{c} \theta_{filtration\;surface}=\theta_{en}\theta_{GBM}\theta_{ep} \end{array}$$ (2)

where *θ*_*en*_, *θ*_*GBM*_ and *θ*_*ep*_ are the solute sieving coefficients through the endothelial cell layer, GBM and the epithelial cell layer, respectively [3]. The calculation of the sieving coefficient across an individual cellular layer is obtained from a steady-state solution of a convection-diffusion equation. The solute diffusivity and convection rate are computed by taking into account the solute-fiber interaction. The detailed calculations of *θ*_*en*_, *θ*_*GBM*_ and *θ*_*ep*_ are discussed extensively in Section C of S1 Appendix.

The hydraulic resistance of the glomerular filtration surface often viewed as the inverse of its hydraulic permeability (1/*k*_*fs*_) is considered to be the resistance of the three layers in series as shown in Eq. (3) as follows.


$$\begin{array}{c} \frac{\text{1}}{k_{fs}}=\frac{\text{1}}{k_{ep}}+\frac{\text{1}}{k_{GBM}}+\frac{\text{1}}{k_{en}} \end{array}$$ (3)

where *k*_*ep*_, *k*_*GBM*_ and *k*_*en*_ are the hydraulic permeability of the epithelial cell layer, that of GBM and that of the endothelial cell layer, respectively [3]; the calculation of the hydraulic permeability of each layer is discussed in Section A of S1 Appendix.

The hydraulic permeability of the four-layered barrier, *k*_*four-layered*_, was computed from a finite element solution of the Laplace equation governing the pressure within the four-layered barrier; the detailed calculation is given in Mathematical Method and in Section B of S1 Appendix. The computation of *θ*_*four-layered*_, the sieving coefficient across the four-layered barrier that includes the glomerular mesangium, was completed by solving the steady-state convection-diffusion equation using the finite element method; the details of the numerical scheme are discussed in Mathematical Method and in Sections D and E of S1 Appendix.

After *k*_*fs*_, *k*_*four-layered*_, *θ*_*filtration surface*_ and *θ*_*four-layered*_ have been determined, the average sieving coefficient, ⟨θ⟩, is computed using Eq. (1); the values of the parameters employed in the calculation are listed in Table 1. The sensitivity of *θ*_*filtration surface*_ to parameters including the GBM thickness, the pressure difference across the glomerular barrier, the osmotic pressure difference and the renal plasma flow rate were examined in our previous work [19]; it was found that other physiological and hemodynamic parameters slightly changed the macromolecule sieving except for the GAG volume fraction in the endothelial fenestrae, *ϕ*_*GAG,en*_, which could significantly alter the macromolecule sieving. To best fitting the experimental data, *ϕ*_*GAG,en*_ was altered manually and slowly until the mean squared error (MSE) between the computed average sieving coefficients, ⟨θ⟩, and the experimental data reached the minimum value. This scheme was only able to minimize the mean squared error between the computed sieving coefficients and those of Ficolls with solute radii, *r*_*s*_, less than or equal to 5 nm. (If *r*_*s*_ exceeded 5 nm, the computed results significantly underestimated the macromolecule sieving coefficients compared to those obtained experimentally using Ficolls.) The value of *ϕ*_*GAG,en*_ that yielded the best fit to the Ficoll sieving coefficients from *in vivo* urinalysis performed in healthy human for *r*_*s*_ ≤ 5 nm was 0.078. If *ϕ*_*GAG,en*_ = 0.078, the total hydraulic permeability of the glomerular filtration surface (*k*_*fs*_) was calculated and found to be 2.38 nm/s/Pa which is within the range of the value that was experimentally obtained at 1.61 – 4.54 nm/s/Pa [30]. For healthy humans, Δ*P* was found to be 36.5 mmHg which is close to the value reported experimentally by Neal et al. [33].

**Table 1:** Physiological and hemodynamic constants employed in the computation of the solute sieving coefficients.

Physiological Parameters and Hemodynamic Factors	Healthy Humans	Patients with Diabetic Nephropathy
GBM thickness, $L_{GBM}$ (nm)	400	800
Fenestrae length, $L_{f}$ (nm)	70	70
Width of a three-layered subunit of the filtration surface, *W* (nm)	500	500
Width of the epithelial slit, *w*_*slit*_ (nm)	43	43
Fraction of GBM surface not covered by the endothelial cells, $\varepsilon_{f}$	0.20	0.20
Fraction of GBM surface not covered by the epithelial podocytes, $\varepsilon_{s}$	0.086	0.086
Number of fenestrae of endothelium in a three-layered subunit, $n_{f}$	3	
Radii of fibers of the slit diaphragm, $r_{c}$ (nm)	6.5	6.5
Radii of type IV collagens, $r_{f}^{collagen}$ (nm)	3.5	3.5
Radii of GAGs, $r_{f}^{GAG}$ (nm)	0.5	0.5
Volume fraction of collagens in GBM, $\phi_{collagen}$	0.05	0.05
Volume fraction of GAGs in GBM, $\phi_{GAG}$	0.05	0.05
GAG volume fraction in the endothelial fenestrae, $\phi_{GAG,en}$	0.078	0.06
Distance between the slit diaphragm and the GBM surface, $L_{GBM-slit}$ (nm)	60	60
Single-nephron glomerular filtration rate, SNGFR (nL/min)	62.5	49.4
Plasma shear viscosity, *μ*_*plasma*_ (mPa.s)	1.2	1.6
Shear viscosity of filtrated fluid in the filtration surface and that in the four-layered barrier, μ (mPa.s)	0.7	0.9
Darcy permeability of the glomerular mesangium, $\kappa_{mes}$ (nm^2^)	14	10
Darcy permeability of GBM, $\kappa_{GBM}$ (nm^2^)	2.2	2.2
Darcy permeability of endothelial fenestrae filled with GAGs, *κ*_*en*_ (nm^2^)	1.53	2.08

**Fig 2:**
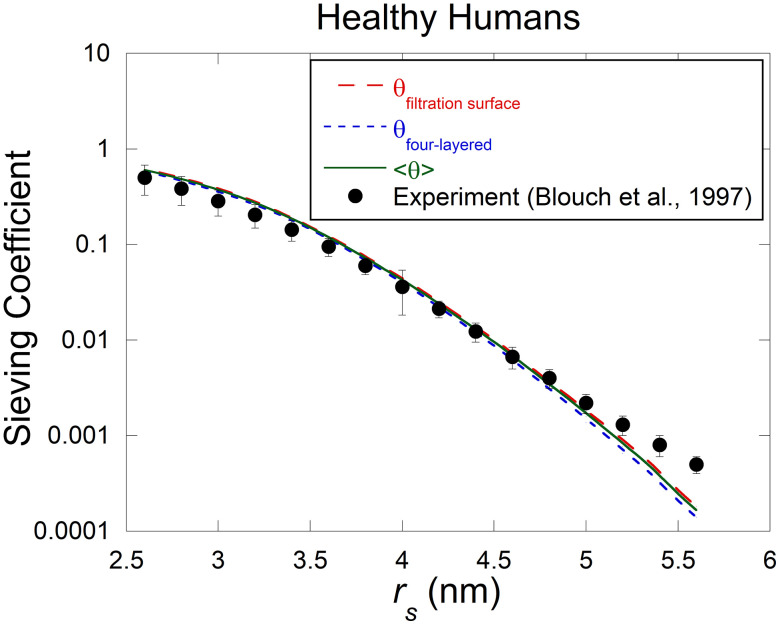
Computed macromolecule sieving coefficient across the filtration surface, *θ*_*filtration surface*_, the sieving coefficient across the four-layered barrier, *θ*_*four-layered*_, and their average, ⟨θ⟩, calculated using Eq. (1) as a function of solute radii, *r*_*s*_. Results were calculated using physiological parameters associated with healthy humans as stated in Table 1. Ficoll sieving coefficients from *in vivo* urinalysis performed in healthy humans [20] are also presented.

The effect of macromolecule transport across the mesangium on the glomerular solute filtration is quite small. In Fig 2, the solute sieving coefficient across the filtration surface (*θ*_*filtration surface*_), that across the four-layered barrier (*θ*_*four-layered*_) and their average value computed as indicated in Eq. (1), ⟨θ⟩, are compared to Ficoll sieving coefficients from urinalysis in healthy humans [20]. As shown in the figure, ⟨θ⟩ only slightly differs from *θ*_*filtration surface*_. *θ*_*four-layered*_ is smaller than *θ*_*filtration surface*_, and, as shown in the figure, the contribution of macromolecule transport across the mesangium to ⟨θ⟩ is found to be small. It is worth noting that both *θ*_*filtration surface*_ and ⟨θ⟩ agree with the experiment if *r*_*s*_ < 5 nm. If *r*_*s*_ exceeds 5 nm, their values are considerably lower than those of Ficoll from *in vivo* urinalysis.

**Fig 3:**
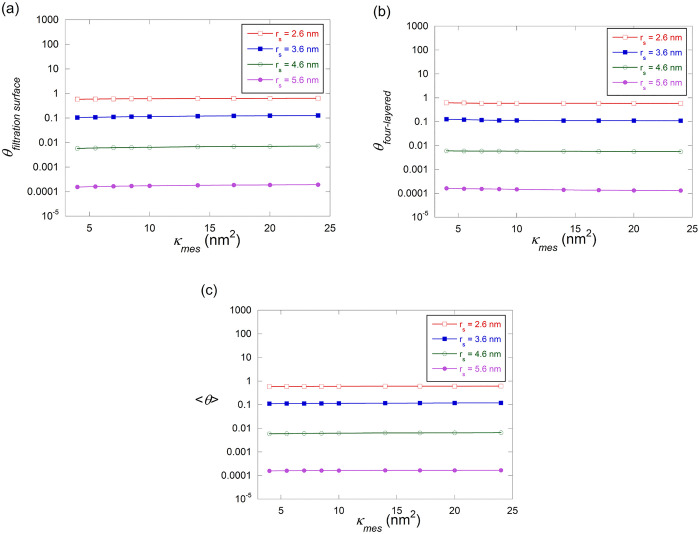
Calculated macromolecule sieving coefficient as a function of the Darcy permeability of the mesangium, *κ*_*mes*_ (nm^2^). Presented results include (a) macromolecule sieving coefficient across the filtration surface, *θ*_*filtration surface*_, (b) the sieving coefficient across the four-layered barrier, *θ*_*four-layered*_, and (c) their average, ⟨θ⟩, calculated using Eq. (1). Results were calculated using physiological parameters associated with healthy humans as stated in Table 1.

Results in Fig 2 is calculated with the mesangial Darcy permeability (*κ*_*mes*_) = 14 nm^2^ which is close to the average value of *κ*_*mes*_ employed by Hunt et al. [27]. To examine the effect of the value of the mesangial Darcy permeability on solute transport, *θ*_*four-layered*_, *θ*_*filtration surface*_ and ⟨θ⟩ are presented as a function of *κ*_*mes*_ in Fig 3a–3c. As shown in Fig 3b, the change in the mesangial Darcy permeability barely alters the sieving coefficient across the four-layered barrier, *θ*_*four-layered*_. *θ*_*filtration surface*_ slightly increases as a function of *κ*_*mes*_ as shown in Fig 3a. This is because the increase in *k*_*four-layered*_ would increase the fraction of fluid volume flow rate across the mesangium, and, therefore, reduce the fraction of the fluid volume flow rate across the filtration surface. In our previous work, *θ*_*filtration surface*_ has been shown to increase if the filtrated fluid velocity decreases [19]. Although, for certain values of *κ*_*mes*_, *θ*_*filtration surface*_ is comparable to *θ*_*four-layered*_, the fraction of the fluid volume flow rate across the filtration surface still exceeds the fraction of the fluid volume flow rate across the four-layered barrier. Therefore, the change of the average sieving coefficient, ⟨θ⟩, as a function of *κ*_*mes*_ is similar to that of *θ*_*filtration surface*_. As shown in Fig 3c, ⟨θ⟩ is hardly affected by the value of *κ*_*mes*_. For results in subsequent figures, the computed solute sieving coefficients were calculated with *κ*_*mes*_ = 14 nm^2^ for healthy humans. For patients with diabetic nephropathy, there is a report of an increase in protein synthesis and accumulation of extracellular proteins such as fibronectin and several types of collagens associated hyperglycemia [34]. As a result, *κ*_*mes*_ was assumed to be slightly lower at 10 nm^2^, although it is worth noting that, as shown in Fig 3c, *κ*_*mes*_ only slightly influences ⟨θ⟩.

**Fig 4:**
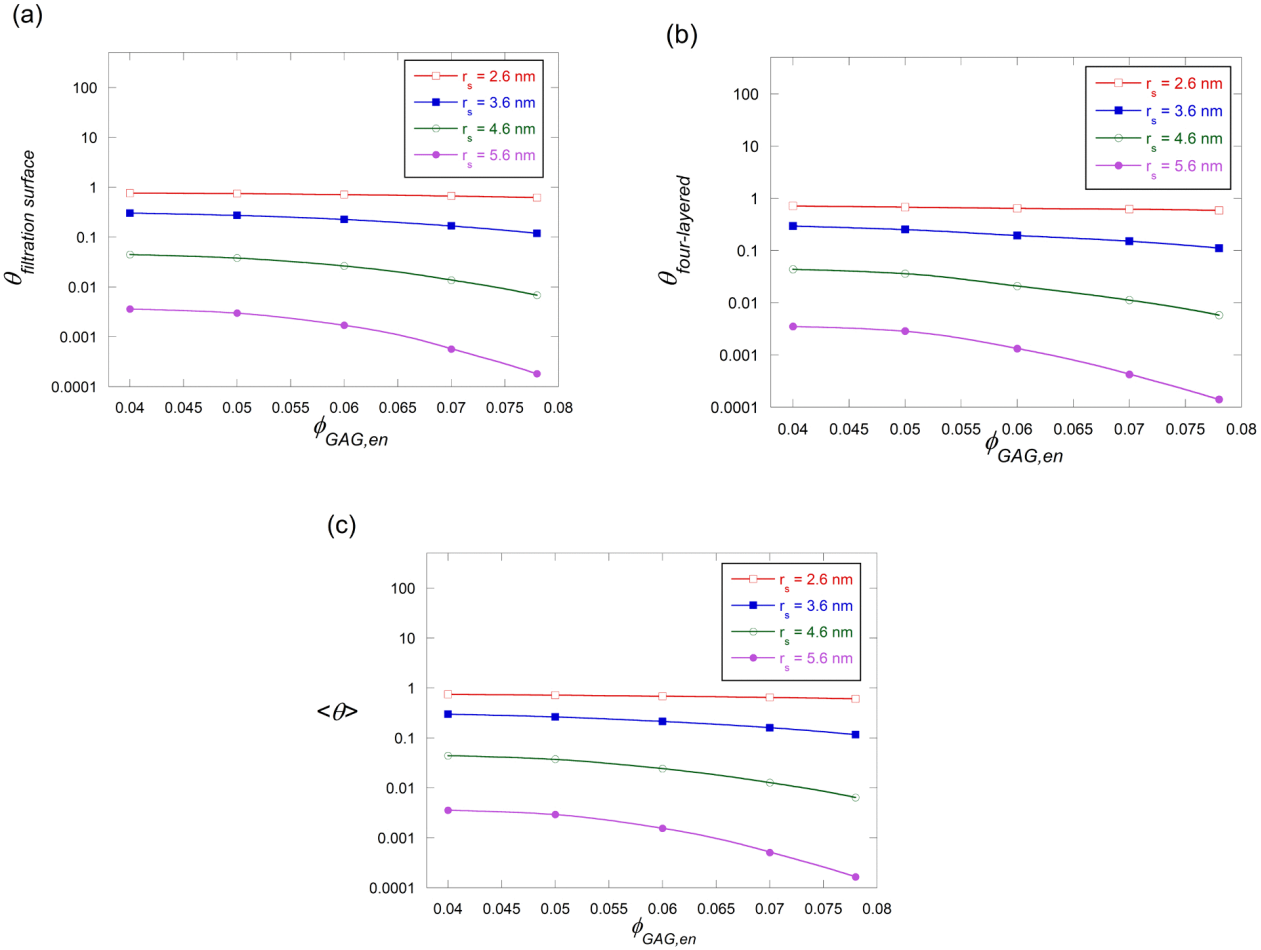
Calculated macromolecule sieving coefficient as a function of GAG volume fraction in the endothelial fenestrae, *ϕ*_*GAG,en*_. Presented results include (a) macromolecule sieving coefficient across the filtration surface, *θ*_*filtration surface*_, (b) the sieving coefficient across the four-layered barrier, *θ*_*four-layered*_, and (c) their average, ⟨θ⟩, calculated using Eq. (1).

Next, the physiological changes associated with nephrotic syndromes affecting glomerular size-selectivity are examined. Because the change in solute sieving through glomerular filtration surface due to GBM thickening is found to be quite small [19], the present study focuses primarily on the effect of the hyperglycemia induced injury resulting in the damage of GAGs in the endothelial fenestrae. In Fig 4a–4c, *θ*_*filtration surface*_, *θ*_*four-layered*_ and their average, ⟨θ⟩, are presented as a function of GAG volume fraction in the endothelial fenestrae (*ϕ*_*GAG,en*_). As shown in the figures, the decrease in *ϕ*_*GAG,en*_ causes an increase in solute sieving; it increases both *θ*_*filtration surface*_ and *θ*_*four-layered*_. For instance, if *r*_*s*_ = 3.6 nm, a reduction of *ϕ*_*GAG,en*_ from 0.078 to 0.06 leads to an approximately twofold increase in *θ*_*filtration-surface*_ and *θ*_*four-layered*_. As shown in Fig 4a–4c, the solute sieving coefficient increase due to the disappearance of GAGs in the endothelial fenestrae is amplified by the increase of the solute radii indicating that GAGs play a vital role in moderate-sized and large glomerular solute restriction. For example, the reduction in *ϕ*_*GAG,en*_ from 0.078 to 0.06 causes a 11% increase in ⟨θ⟩ if *r*_*s*_ = 2.6 nm. If *r*_*s*_ = 5.6 nm, however, ⟨θ⟩ computed with *ϕ*_*GAG,en*_ being 0.078 is approximately an order of magnitude smaller than that calculated with *ϕ*_*GAG,en*_ being 0.06.

**Fig 5:**
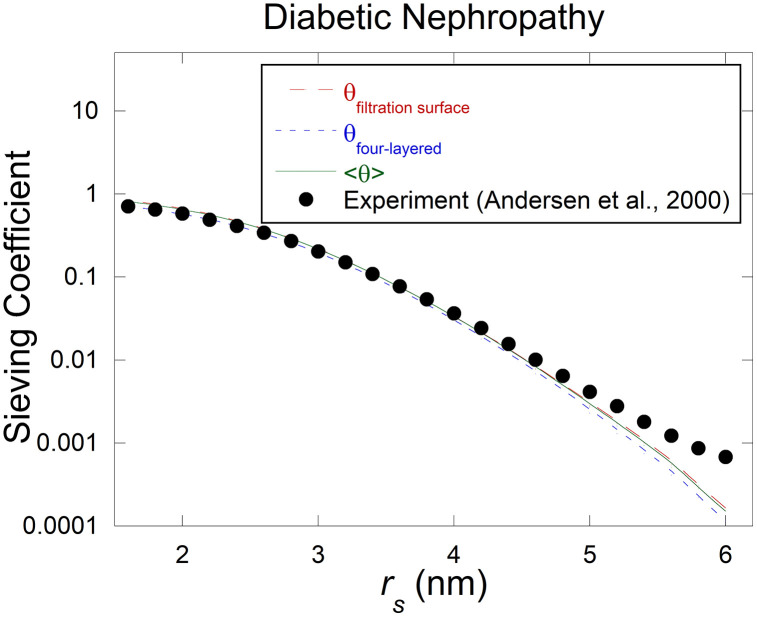
Computed macromolecule sieving coefficient across the filtration surface, *θ*_*filtration surface*_, the sieving coefficient across the four-layered barrier, *θ*_*four-layered*_, and their average, ⟨θ⟩, as a function of solute radii, *r*_*s*_. Results were calculated using physiological parameters associated with patients with diabetic nephropathy as stated in Table 1. Also presented are Ficoll sieving coefficients from *in vivo* urinalysis performed in patients with early-but-overt diabetic nephropathy [21].

The contribution of the glomerular mesangium to solute restriction in patients with diabetic nephropathy is illustrated in Fig 5 where *θ*_*filtration surface*_, *θ*_*four-layered*_ and ⟨θ⟩ are presented as a function of *r*_*s*_, and compared to Ficoll sieving coefficients from urinalysis performed in patients with early-but-overt diabetic nephropathy [21]. *θ*_*filtration surface*_, *θ*_*four-layered*_ and ⟨θ⟩ were computed by using the physiological and hemodynamic factors for patients with diabetic nephropathy stated in Table 1 where the GBM thickness was increased to 800 nm. The best fit was obtained when the GAG volume fraction in the endothelial fenestrae was assumed to be slightly reduced due to hyperglycemia down to 0.06. As shown in the figure, *θ*_*four-layered*_ is close to *θ*_*filtration surface*_, but as the flux through the filtration surface is larger than that through the four-layered barrier, the contribution of *θ*_*four-layered*_ to ⟨θ⟩ is still small. A trend similar to that of the sieving coefficient computed for healthy humans is observed; ⟨θ⟩ (calculated by employing the physiological and hemodynamic parameters of patients with diabetic nephropathy) agrees with Ficoll sieving coefficients if *r*_*s*_ ≤ 5 nm. For larger *r*_*s*_, the computed ⟨θ⟩ is found to be smaller than the sieving coefficients of Ficolls from experiments with the underestimation increases as a function of the solute radii.

### Effects of solute transport through shear-induced shunts on glomerular size-selectivity

In this section, the effect of the presence of shear-induced shunts on glomerular macromolecule filtration is investigated. The comparison between our computed results and the sieving coefficients of Ficolls obtained from urinalysis indicates that, if only the sole effect of solute transport across the filtration surface and that through the four-layered barrier are included in the calculation, the computed results agree very well with the experimental data for *r*_*s*_ up to 5 nm, but the calculation yields the solute sieving coefficients that are smaller than Ficoll sieving coefficients if *r*_*s*_ exceeds 5 nm. This indicates that the mesangial matrix is not the pathway for transporting large macromolecules and could not be the origin of the “diminishing glomerular size-selectivity” when *r*_*s*_ > 5 nm. As aforementioned, the micrographs showing RBCs escaping through a gap at the junction between the filtration surface and the four-layered barrier and the evidence of RBCs found in urine lead us to speculate that there are possibly shunts at the junction between the filtration surface and the four-layered barrier created by a shear flow due to the velocity difference of the fluid flowing through the filtration surface and that transported through the mesangium that may change the orientations of collagens within GBM. In the present study, it is assumed that the shunts open and close periodically as a function of time because of the images from microscopy of Collar et al. [28] and Liapis et al. [29]. The shunt radius, *R*(*t*), is, *t*herefore a function of time. If the shunts gradually open and close; $R\left(t\right)=R_{\text{0}}sin\left(\pi t/\tau \right)$ with *R*_0_ being the maximum radius of the shunt and τ being the shunt opening and closing period as indicated in Eq. (12) and discussed further in Mathematical Method. The total sieving coefficient that includes the fluxes across the filtration surface, the mesangial area and the shear-induced shunt, $\left\langle \theta_{total}\right\rangle$, is computed using Eq. (4) as shown below.


$$\begin{array}{c} \left\langle \theta_{total}\right\rangle =\frac{C_{B}}{C_{\text{0}}}=\left\langle \theta \right\rangle +\frac{\left\langle R_{v}\right\rangle \left\langle \theta_{shunt}\right\rangle }{\text{SNGFR}} \end{array}$$ (4)

where $\left\langle \theta_{shunt}\right\rangle$ is the average sieving coefficient through the periodically opening and closing shunts. Its computation is discussed in Mathematical Method and in Section F of S1 Appendix. $\left\langle R_{\nu }\right\rangle$ is the average fluid flow rate transported through the shunt; the detailed calculation is given in Mathematical Method. The employed parameters are listed in Table 1.

**Fig 6:**
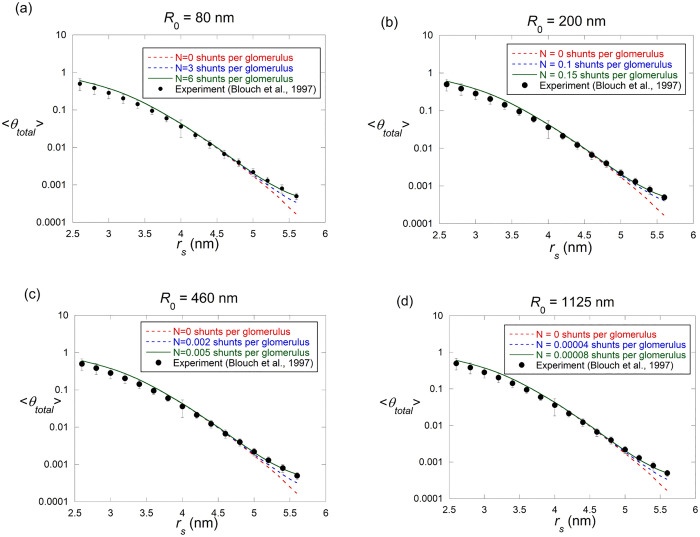
Total macromolecule sieving coefficient, $\left\langle \theta_{total}\right\rangle$, calculated from the solute fluxes across the filtration surface, the four-layered barrier and the shear induced shunts at the junction between the two barriers using Eq. (4). *R*_0_ is the maximum shunt radius during the periodic opening and closing of the shunt. Presented results include $\left\langle \theta_{total}\right\rangle$ computed with **(a)** *R*_0_ = 80 nm, **(b)** *R*_0_ = 200 nm, **(c)** *R*_0_ = 460 nm and **(d)** *R*_0_ = 1125 nm. *N* is the average number of shunts per glomerulus. (For example, *N* = 0.00008 shunts per glomerulus means that there are 8 shunts per 100,000 glomeruli at a time.) Results were computed using physiological parameters associated with healthy humans as stated in Table 1 and are presented as a function of solute radii, *r*_*s*_. Also included are Ficoll sieving coefficients from *in vivo* urinalysis performed in healthy humans [20].

The effect of the solute filtration through the possibly shear-induced shunts is illustrated in Fig 6a–6d where the total sieving coefficient, $\left\langle \theta_{total}\right\rangle$, computed from the solute fluxes through the filtration surface, the four-layered, and the shunts into Bowman’s Space as indicated in Eq. (4), is presented as a function of *r*_*s*_. Four possible values of the maximum radius of the shunt during its periodic opening and closing (*R*_0_) are examined; in Fig 6a, *R*_0_ = 80 nm based on the reported micrographs of Liapis et al. [29]. In Fig 6d, *R*_0_ = 1.125 µm, the maximum radius reported by Collar et al. [28]. As the subject of the work of Collar et al. had thin GBM, we consider *R*_0_ = 1.125 µm to be the upper bound value for *R*_0_. In addition, Fig 6b and 6c show computed results where *R*_0_ = 200 nm and 460 nm, the maximum pore radii based on the observation of Neal and Michel [35] of pores created by a rupture in frog vessels cited by Collar et al. [28] as the only other known system where a rupture creating pores through which RBCs escape was followed by a recovery. Also presented is the Ficoll sieving coefficients obtained from *in vivo* urinalysis in healthy humans [20]. *N* is the average number of shunts per glomerulus. To best fitting the experimental data, the value of *N* was increased manually and slowly until the mean squared error (MSE) between the computed total sieving coefficient, $\left\langle \theta_{total}\right\rangle$, and the experimental data reached its minimum value with data points being throughout the entire range of presented solute radii.

As shown in all of the figures, if the solute radius is small with *r*_*s*_ < 5.0 nm, the effect of the presence of the shunts on the solute sieving coefficient is found to be small. The results computed with all values of *N* are close to that of *N* = 0. If *r*_*s*_ exceeds 5.0 nm, however, the difference of the computed sieving coefficients due to the differing values of *N* becomes graphically distinguishable, indicating that the effect of the presence of the shunts on glomerular solute filtration is stronger for large solutes. It is true that the average solute sieving coefficient through the shunt, $\left\langle \theta_{shunt}\right\rangle$, is close to 1 and larger than the average solute sieving coefficient computed from the solute flux through filtration surface and that across the four-layered barrier, ⟨θ⟩. However, they are still in the same order of magnitude or ⟨θ⟩ is a few orders of magnitude smaller than $\left\langle \theta_{shunt}\right\rangle$ if *r*_*s*_ < 5 nm. The average fluid volume flow rate through the shunts, $\left\langle R_{v}\right\rangle$, is, on the other hand, much smaller than the volume flow rate through the filtration surface and the four-layered barrier. For instance, if *R*_0_ = 80 nm, SNGFR is not altered by the presence of the shunts as long as *N* ≤ 30 shunts per glomerulus. For other values of *R*_0_, the fraction of fluid flux passing through the shunts (if *N* are the values that yield the best fit to experimental data) remains very small. This results in $\left\langle \theta_{total}\right\rangle$ being graphically indistinguishable from ⟨θ⟩ for small and medium-sized solutes with *r*_*s*_ < 5 nm. As *r*_*s*_ increases and exceeds 5 nm, however, ⟨θ⟩ becomes very small such that the last term on the right-hand side of Eq. (4) becomes comparable to the other terms. The presence of the shunts, therefore, strongly affects $\left\langle \theta_{total}\right\rangle$ of solutes with *r*_*s*_ > 5 nm, but barely changes the total sieving coefficients of smaller solutes. It is also worth noting that the values of *N* that yield the best fit to the Ficoll sieving coefficients from urinalysis studies [20] decrease as a function of *R*_0_. *N* = 6 shunts per glomerulus yields the best fit if *R*_0_ = 80 nm, whereas, if *R*_0_ = 200, 460 and 1125 nm, *N* yielding the best fit are 0.15, 0.005 and 0.00008 shunts per glomerulus, respectively. (*N* is considered to be an average number of shunts per glomerulus at a time. For instance, *N* = 0.00008 shunts per glomerulus means that there are 8 shunts per 100,000 glomeruli at a time.) For all employed values of *R*_0_ (if *N* are the values that yield the best fit to the experimental data), MSE is 0.0023, and, accordingly, the root mean squared error (RMSE) = 0.048. Despite the differing values of *N* yielding the best fit, the main conclusion from Fig 6a–6d remains that the possibly shear induced shunt at the junction between the filtration surface and the four-layered barrier is possibly the main pathway for transport of large macromolecules.

**Fig 7:**
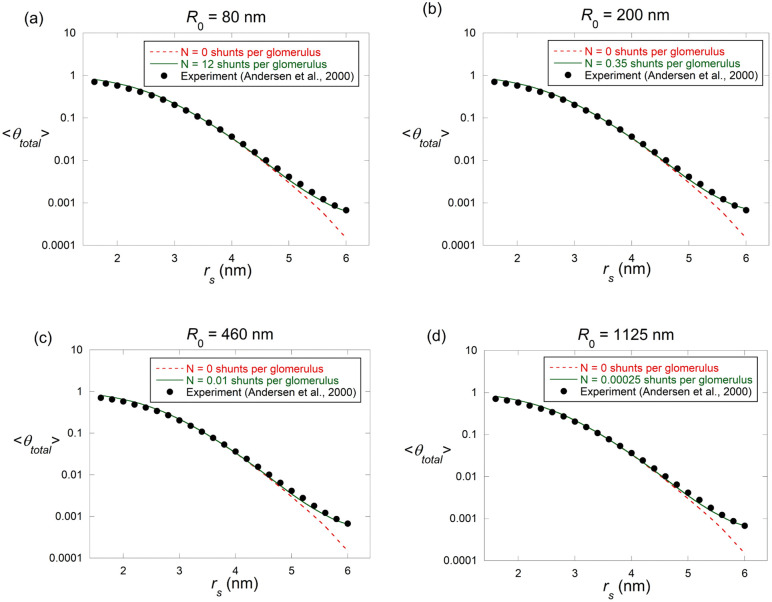
Total macromolecule sieving coefficient, $\left\langle \theta_{total}\right\rangle$, calculated from the solute fluxes across the filtration surface, the four-layered barrier and the shear induced shunts at the junction between the two barriers using Eq. (4). *R*_0_ is the maximum shunt radius during the periodic opening and closing of the shunt. Presented results include $\left\langle \theta_{total}\right\rangle$ computed with **(a)** *R*_0_ = 80 nm, **(b)** *R*_0_ = 200 nm, **(c)** *R*_0_ = 460 nm and **(d)** *R*_0_ = 1125 nm. *N* is the average number of shunts per glomerulus. Results were computed using physiological parameters associated with patients with diabetic nephropathy as stated in Table 1 and are presented as a function of solute radii, *r*_*s*_. Also included are Ficoll sieving coefficients from *in vivo* urinalysis performed in patients with diabetic nephropathy [21].

To examine the possible effect of the presence of the shunts on glomerular size-selectivity in patients with diabetic nephropathy, $\left\langle \theta_{total}\right\rangle$ computed by employing the physiological parameters and hemodynamic factors from observations performed in patients with diabetic nephropathy as indicted in Table 1 is presented as a function of the solute radii (*r*_*s*_) and compared to Ficoll sieving coefficients from urinalysis in patients with early-but-overt diabetic nephropathy [21] in Fig 7a–7d where *R*_0_ = 80 nm, 200 nm, 460 nm and 1.125 µm, respectively. Because of hyperglycemia, the plasma viscosity (*μ*_*plasma*_) is assumed slightly higher than the plasma viscosity of healthy humans and set at 1.6 mPa.s. If *R*_0_ = 80 nm, the value of *N* that yields the computed $\left\langle \theta_{total}\right\rangle$ that agrees well with the experimentally obtained Ficoll sieving coefficients for *r*_*s*_ = 1.6 – 6 nm is 12 shunts per glomerulus. For *R*_0_ = 200 nm, 460 nm and 1125 nm, *N* yielding the best fit are 0.35, 0.01 and 0.00025 shunts per glomerulus, respectively. This is similar to the trend seen in Fig 6a–6c; *N* that yields the best fit with Ficoll sieving coefficients from *in vivo* studies declines as a function of *R*_0_. MSE for all values of *R*_0_ (if *N* are the values yielding the best fit) is 0.0012 and RMSE is, as a result, 0.035. Fig 7a–7d demonstrate that, in patients with diabetic nephropathy, the shunts at the junction is still possibly the main pathway for large solute transport.

**Fig 8:**
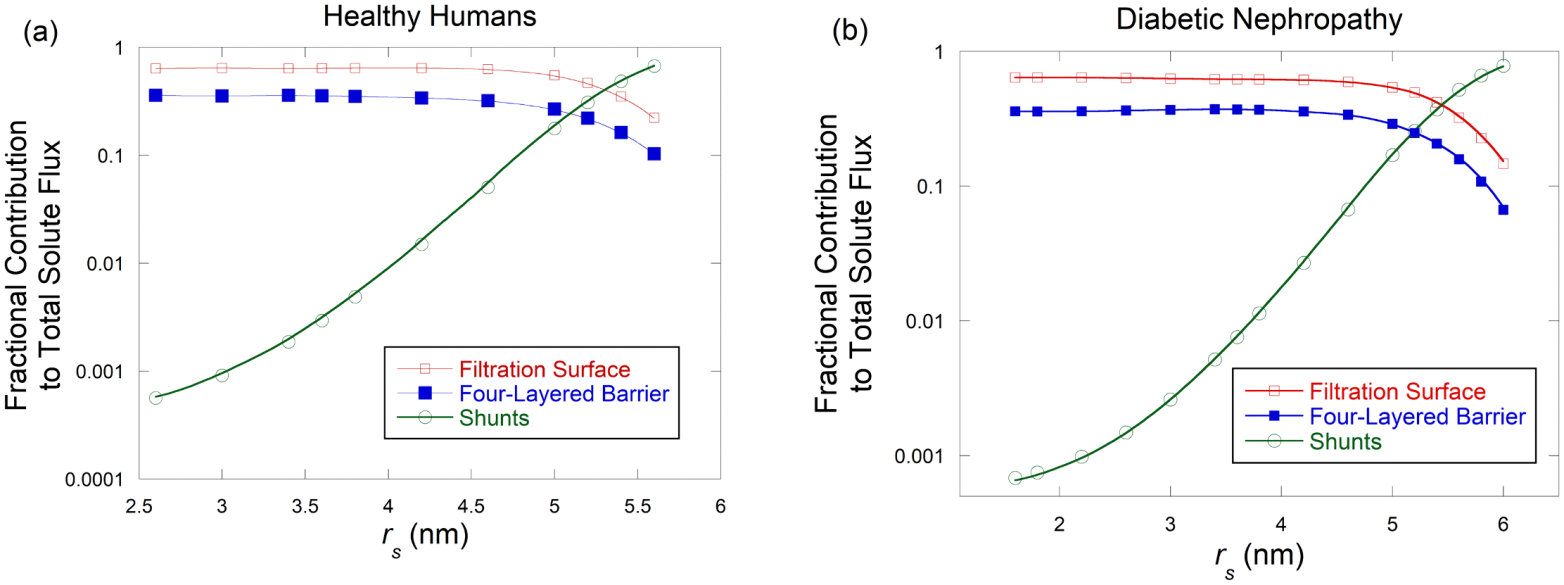
Fractional contribution to total solute flux of the solute flux across the filtration surface, the solute flux across the four-layered barrier, and the solute flux through the shear induced shunts at the junction between the two barriers presented as a function of solute radii, *r*_*s*_. *R*_0_, the maximum shunt radius during the periodic opening and closing of the shunt, is 80 nm. *N* is the average number of shunts per glomerulus. Results were computed by (a) using physiological parameters associated with healthy humans with *N* = 6 shunts per glomerulus and (b) using the physiological parameters associated with patients with diabetic nephropathy with *N* = 12 shunts per glomerulus.

In order to examine the relative contribution of the three pathways to glomerular size-selectivity, fractional contribution to total solute flux of the solute flux across the glomerular filtration surface, that of the solute flux across the four-layered barrier and that of the solute flux through the shunt at the junction are plotted as a function of solute radii (*r*_*s*_) in Fig 8a where the solute fluxes were computed using parameters associated with healthy humans in Table 1. *R*_0_ = 80 nm and *N* = 6 shunts per glomerulus. (Even though it is not shown here, the same trend is observed for *R*_0_ = 200 nm, 460 nm or 1125 nm with *N* being the values yielding the best fit to the experiment. The results can be accessed through the DOI given in Data Availability.) As shown in the figure, for *r*_*s*_ < 5 nm, the main pathway is across the filtration surface with the contribution to solute flux through the shunts being negligible. As *r*_*s*_ exceeds 5 nm, the fractional contribution of the solute flux through the shunts becomes comparable to the fractional contributions of the other two pathways and eventually becomes larger at *r*_*s*_ > 5.5 nm. The trend is also evident in Fig 8b where the fractional contributions to solute flux of the three pathways were computed using parameters associated with patients with diabetic nephropathy in Table 1. *R*_0_ = 80 nm and *N* = 12 shunts per glomerulus. At *r*_*s*_ = 6 nm, the fractional contribution to the total transcapillary solute flux of the solute flux through the shunts at the junction is 0.786, whereas the fractional contribution of the solute flux across the filtration surface and that of the solute flux across the four-layered barrier decline to 0.147 and 0.067, respectively. We believe it is the first time that the fractional contributions to solute flux of solute transport via the three pathways are compared; results indicate that, for glomerular size-selectivity, the solute size may possibly determine the main solute transport pathway. For small and moderate-sized solute, most of the solute flux flows across the glomerular filtration surface, whereas the shunts at the junction are the main pathway for large macromolecule sieving.

## Discussion

Our simulation results indicate that the inclusion of solute transport through the shear-induced shunts at the junction between the glomerular filtration surface and the glomerular mesangium yields sieving coefficients of large macromolecules that agree with experimental data when *r*_*s*_ exceeds 5 nm. Although the presence of the shunts hardly affects passages of small and moderate sized macromolecules, it increases the sieving coefficients of large solutes significantly. This indicates that there can be different major pathways for macromolecules of differing sizes. For small and moderate-sized solutes, the major pathway for solute passages is the glomerular filtration surface, whereas for large macromolecules, the shear-induced shunts at the junction between the glomerular filtration surface and the glomerular mesangium can serve as the main transport pathway. The shear-induced shunts may also help explain the “upper limit” in glomerular size-selectivity as to why the sieving coefficient of Ficolls with *r*_*s*_ = 5 nm is not very different from that of Ficolls with *r*_*s*_ = 8 nm as observed from urinalysis performed in sheep [22].

The significant elevation of sieving coefficients of large Ficolls from that of healthy humans has been observed in subjects with nephrotic syndromes such as membranous nephropathy [20]. Sieving coefficients of small Ficolls in patients with membranous nephropathy are slightly smaller than those in healthy humans, while sieving coefficients of large Ficolls in patients with membranous nephropathy are considerably larger than those in healthy humans. For example, the average sieving coefficient of Ficolls with *r*_*s*_ = 3.6 nm (the same size as the Stokes-Einstein radius of serum albumins) in patients with membranous nephropathy is approximately 7% lower than that from the same study performed in healthy humans [20]. On the other hand, for Ficolls with *r*_*s*_ = 5.6 nm, the average sieving coefficient in patients with membranous nephropathy is found to be two orders of magnitudes larger than that in healthy humans. Increasing transport through the shear-induced shunts (due to physiological alterations and pathological changes associated with renal diseases) may partially explain the selective amplification of glomerular passage of large macromolecules. Testing this hypothesis is one of the possible directions for future works.

### Model limitations

**Fig 9:**
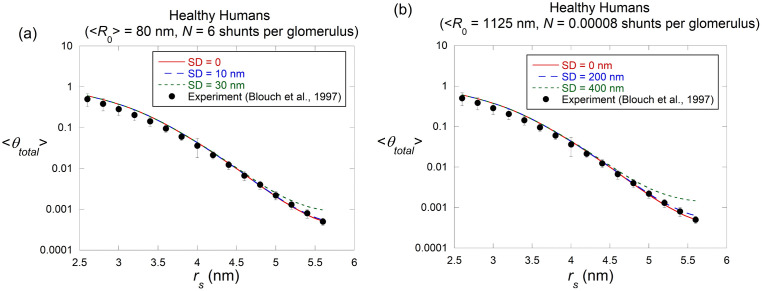
Total macromolecule sieving coefficient, $\left\langle \theta_{total}\right\rangle$, calculated by assuming that the maximum shunt radius during the periodic opening and closing of the shunts, *R*_0_, follows the lognormal distribution with <*R*_0_ > being the mean value of *R*_0_ and SD being its standard deviation. *N* is the average number of shunts per glomerulus. Results are computed with **(a)** <*R*_0_ > = 80 nm and *N* = 6 shunts per glomerulus and **(b)** <*R*_0_ > = 1125 nm and *N* = 0.00008 shunts per glomerulus. Results were computed using physiological parameters associated with healthy humans as stated in Table 1 and are presented as a function of solute radii, *r*_*s*_. Also included are the Ficoll sieving coefficients from *in vivo* urinalysis performed in healthy humans [20].

**Fig 10:**
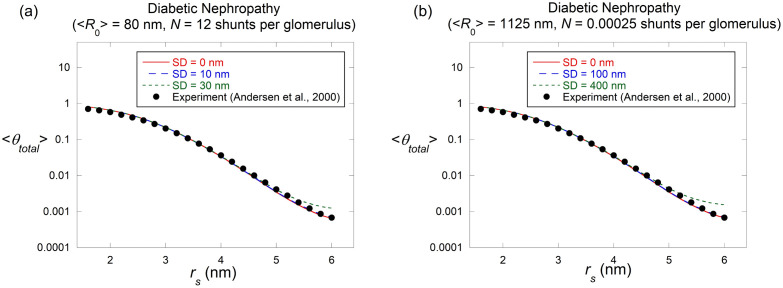
Total macromolecule sieving coefficient, $\left\langle \theta_{total}\right\rangle$, calculated by assuming that the maximum shunt radius during the periodic opening and closing of the shunt, *R*_0_, follows the lognormal distribution with <*R*_0_ > being the mean value of *R*_0_ and SD being its standard deviation. *N* is the average number of shunts per glomerulus. Results were computed with **(a)** <*R*_0_ > = 80 nm and *N* = 12 shunts per glomerulus and **(b)** <*R*_0_ > = 1125 nm and *N* = 0.00025 shunts per glomerulus. Results were computed using physiological parameters associated with patients with diabetic nephropathy as stated in Table 1 and are presented as a function of solute radii, *r*_*s*_. Also included are the Ficoll sieving coefficients from *in vivo* urinalysis performed in patients with diabetic nephropathy [21].

It is also worth noting that the value of *R*_0_ have a strong effect on $\left\langle \theta_{total}\right\rangle$ as shown in Fig 6a–6c as well as Fig 7a–7d; while the change in $\left\langle \theta_{total}\right\rangle$ caused by the variation of *R*_0_ is very small for small and moderate solutes, the decrease in *N* yielding the sieving coefficients that agree well with those of Ficolls from urinalysis study as a function of *R*_0_ is evident especially for larger solutes, emphasizing the fact that the presence of the shear-induced shunts strongly affects large macromolecule passages. Because of the biological variability, the shunt radius, *R*(*t*), with a single value of *R*_0_ may not be realistic. To examine the effect of shunt radius heterogenei*t*y, *R*_0_ is assumed to follow a lognormal distribution. (The numerical scheme is described in Section G of S1 Appendix.) As shown in Fig 9a and 9b, < *R*_0_ > , the mean value of *R*_0_, are set at 80 nm and 1125 nm, where *N* = 6 shunts per glomerulus and 0.00008 shunts per glomerulus, respectively. The standard deviation of the distribution of *R*_0_ (SD) is varied. Results indicate that the increase in SD increases the sieving coefficient. Computed results shown in Fig 10a and 10b demonstrates that the same trend is observed for patients with early-but-overt diabetic nephropathy. As SD is currently undetermined, *N* yielding the best fit to the experimental data computed by using a single value of *R*_0_ should be considered the upper bound estimate (because, if SD ≠ 0, *N* yielding the best fit could be lower). The value of shunt radii requires further investigation and is one of the limitations of our current mathematical model.

**Fig 11:**
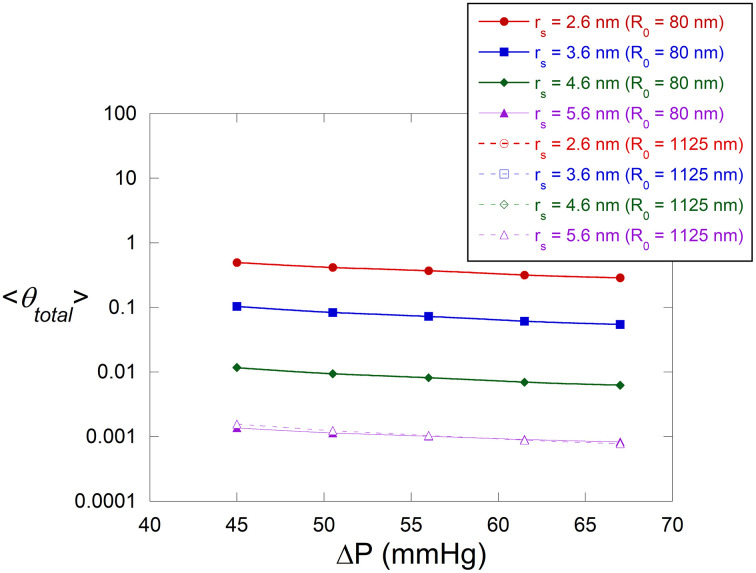
Total macromolecule sieving coefficient, $\left\langle \theta_{total}\right\rangle$, as a function of the hydraulic pressure difference across the glomerular barrier, ΔP. *R*_0_ is the maximum shunt radius during the periodic opening and closing of the shunt, whereas *N* is the average number of shunts per glomerulus. Results are calculated with *R*_0_ = 80 nm and *N* = 12 shunts per glomerulus as well as with *R*_0_ = 1125 nm and *N* = 0.00025 shunts per glomerulus. Results were computed using physiological parameters associated with patients with diabetic nephropathy as stated in Table 1.

In addition, although the employed hydraulic pressure difference estimated from SNGFR of healthy humans is close to the value reported from an experiment [33], Δ*P* for patients with diabetic nephropathy remains unknown and is currently calculated from SNGFR using Eq. (14); the experimental confirmation of Δ*P* in patients with diabetic nephropathy is desired. To shed some light on the effect of the uncertainty of Δ*P*, $\left\langle \theta_{total}\right\rangle$ for patients with diabetic nephropathy is plotted as a function of Δ*P* in Fig 11. Results shown that $\left\langle \theta_{total}\right\rangle$ declines as Δ*P* increases. If the hydraulic permeability of the filtration surface and that of the four-layered barrier as well as other parameters are kept constant, the increase in Δ*P* causes an increase in SNGFR. If the value of glomerular filtration rate, GFR, is constant, a larger value of Δ*P* corresponds to the smaller number of nephrons. The values of GFR for patients with diabetic nephropathy from the experiment of Andersen et al. [21] is reported to be 79 mL/min. Results shown in Fig 11 correspond to the total number of nephrons per kidney being 0.59 – 1.27 million nephrons.

**Fig 12:**
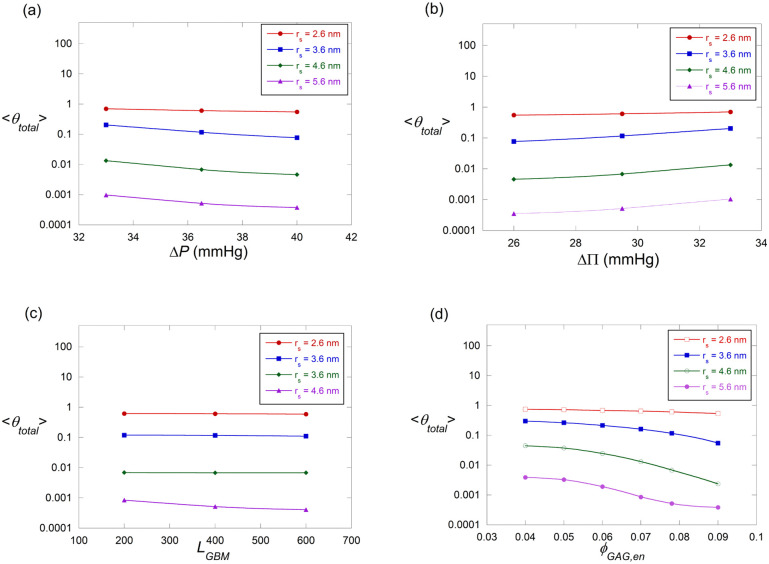
Total macromolecule sieving coefficient, $\left\langle \theta_{total}\right\rangle$, presented as a function of (a) the hydraulic pressure difference across the glomerular barrier (ΔP), (b) the osmotic pressure difference across the glomerular barrier (ΔΠ), (c) the GBM thickness (*L*_GBM_) and (d) the GAG volume fraction in the endothelial fenestrae $\left(\phi_{GAG,en}\right)$. *R*_0_ is the maximum shunt radius during the periodic opening and closing of the shunt, whereas *N* is the average number of shunts per glomerulus. Results were calculated with *R*_0_ = 80 nm and *N* = 6 shunts per glomerulus by using physiological parameters associated with healthy humans as stated in Table 1.

As shown in Fig 3c, the variation in the mesangial Darcy permeability ($\kappa_{mes}$) only slightly changes ⟨θ⟩. As a result, it hardly alters $\left\langle \theta_{total}\right\rangle$. For instance, if *r*_*s*_ = 3.6 nm (the Stokes-Einstein radius of serum albumin), the six fold increase in $\kappa_{mes}$ results in the 8% change in the total sieving coefficient. The uncertainty in $\left\langle \theta_{total}\right\rangle$ in healthy humans caused by the uncertainty of other key inputs is shown in Fig 12a – 12d where $\left\langle \theta_{total}\right\rangle$ is plotted as a function of the transcapillary pressure difference (Δ*P*), the transcapillary osmotic pressure difference (ΔΠ), the GBM thickness $\left(L_{GBM}\right)$ and the GAG volume fraction in the endothelial fenestrae $\left(\phi_{GAG,en}\right)$, respectively. *R*_0_ = 80 nm and *N* = 6 shunts per glomerulus. As indicated in Fig 12a, $\left\langle \theta_{total}\right\rangle$ declines as Δ*P* increases, but it increases as a function of ΔΠ as shown in Fig 12b. This is similar to the trend of the sieving coefficient declining as a function of the local fluid velocity shown in our previous work [19]. The effect of GBM thickening is interesting; if *r*_*s*_ = 2.6 nm, 3.6 nm or 4.6 nm, the increase in *L*_*GBM*_ hardly affects $\left\langle \theta_{total}\right\rangle$, but it evidently decreases the total sieving coefficient if *r*_*s*_ = 5.6 nm. This is due to the fact that GBM thickening almost does not change $\theta_{filtration\;surface}$. However, it decreases the solute transport through shunts with the effect becoming significant when *r*_*s*_ exceeds 5 nm, indicating that the shunts may possibly be the main pathway for large solutes. This is similar to the change in $\left\langle \theta_{total}\right\rangle$ due to the variation of GAG volume fraction in the endothelial fenestrae ($\phi_{GAG,en}$) shown in Fig 12d. If *r*_*s*_ = 2.6 nm, 3.6 nm or 4.6 nm, $\left\langle \theta_{total}\right\rangle$ declines as a function of $\phi_{GAG,en}$ similarly to the change in ⟨θ⟩ shown in Fig 4c. If *r*_*s*_ = 5.6 nm, the competing effect between ⟨θ⟩ and $\left\langle \theta_{shunt}\right\rangle$ on $\left\langle \theta_{total}\right\rangle$ is observed.

Another limitation of the present work is that it focuses on size-selectivity while not exploring the charge effects which is another direction of future work. We believe that electrostatic effects are likely to hinder solute transport. The sieving coefficients of charged proteins across the glomerular capillary wall (measured using various techniques) are lower than those of neutral Ficolls of comparable size [1]. Nephrotic syndromes such as membranous nephropathy elevate the clearance of proteins such as serum albumins and immunoglobulin G (relative to that of healthy subjects) by 2–3 orders of magnitudes [20]. This could potentially be due to the deformity of the components contributing to charge-selectivity such as the damage of GAGs. However, it is worth nothing that the sieving coefficients of large neutral Ficolls are also elevated in subjects under the same nephrotic syndrome by several orders of magnitude (relative to those of healthy humans) indicating that the increase in *N* or *R*_0_ may also contribute to the large increase in protein clearance.

As our computed results are compared to the Ficoll sieving coefficients, Ficoll polydispersity is one of the limitation of the present work. Directions of future work also include investigations of the contribution of strain rate and shear stress associated with renal diseases [36,37] and the effect on fluid filtration due to subpodocyte areas [38] on the glomerular fluid and macromolecule transport.

## Mathematical method

As shown schematically in Fig 1, in the present study, the glomerular capillary wall is assumed to consist of three components: the filtration surface, the four-layered barrier with the mesangium located between the endothelial cell layer and GBM, and the shunts at the junction between the filtration surface and the four-layered barrier that open and close periodically. The objective of the present work is to estimate the relative contribution of each component to glomerular size-selectivity. Solutes are assumed to be uncharged rigid spherical particles large enough to be considered hydrodynamic particles. The mathematical method employed in our calculation is discussed below, starting with the calculation of the fluid flux across the glomerular filtration surface, the mesangium and the shear-induced shunt at the junction between the filtration surface and the four-layered barrier. The computation of solute transport across the filtration surface, the four-layered barrier and through the shunts at the junction are also discussed. Finally, the numerical scheme involving the average sieving coefficient as well as the employed parameters are presented.

### Fluid transport across the glomerular capillary wall

In our calculation, the fluid from the circulation is filtered through the glomerular filtration surface, the four-layered barrier that includes the mesangium and the shunts at the junction between these two barriers. The flow direction is indicated in Fig 1, and the computation of the fluid flux across the individual layer is discussed below.

#### Hydraulic permeability of the glomerular filtration surface.

In the calculation of the fluid flux across the glomerular filtration surface, the ultrastructural model [12] was employed where the filtration surface consisted of repeating subunits along the length of the capillary. The fluid is transported from the lumen through the endothelial fenestrae, across GBM and the slit diaphragm into Bowman’s Space [1]. The hydraulic permeability of the slit diaphragm, *k*_*ep*_, was computed as the average velocity of the fluid filtrated through a row of parallel cylinders with non-uniform spacing per unit pressure difference. GBM was assumed to be a hydrogel containing two types of fibers with differing sizes and these fibers strongly influenced the fluid transport across the basement membrane. The endothelial fenestrae were assumed to be filled with GAGs. The presence of the fibers, especially GAGs, strongly affected the fluid flow permeability [39,40]. The calculation of *k*_*ep*_, *k*_*GBM*_ and *k*_*en*_ are discussed in details in Section A of S1 Appendix. The hydraulic permeability of the glomerular filtration surface was, then, calculated using Eq. (3). The computed value of *k*_*fs*_ along with that of the hydraulic permeability of the four-layered surface and those of the shunts at the junction between the filtration surface and the four-layered barrier were employed in determining the hydraulic pressure difference across the glomerular capillary wall from the reported value of GFR.

#### Fluid filtration through the four-layered barrier.

As aforementioned, a part of the glomerular capillary wall is the four layered barrier where the fluid is transported through the endothelial fenestrae, the glomerular mesangium, GBM and the epithelial cell layer as shown schematically in Fig 1. Following the approach previously employed by Hunt et al. [27], the fluid velocity (**v**_*mesangium*_) and the local pressure in the glomerular mesangium (*P*) are related through Darcy’s law as shown below.


$$\begin{array}{c} {𝐯}_{mesangium}=-\frac{\kappa_{mes}}{\mu }\nabla P \end{array}$$ (5)

where *κ*_*mes*_ is the Darcy permeability of the glomerular mesangium. A substitution of the above expression in to the continuity equation yield the governing equation $\left(\nabla \cdot {𝐯}_{mesangium}=0\right)$ for *P* as follows.


$$\begin{array}{c} \nabla^{\text{2}}P=0 \end{array}$$ (6)

Similarly, by following the formulation of Drumond and Deen (1994a), the relationship of the local fluid velocity (**v**_*GBM*_) and the local pressure in GBM can also be described using Darcy’s law, Eq. (5), but with the Darcy permeability being *κ*_*GBM*_, the GBM Darcy permeability. Substituting the expression for **v**_*GBM*_ into the continuity equation $\left(\nabla \cdot {𝐯}_{GBM}=0\right)$ yields the Laplace equation, Eq. (6), as the governing equation for the local pressure in GBM. As for the fluid filtration through the endothelial fenestrae, under an assumption that the contribution to fluid transport restriction of contained GAGs [39,40] i was much larger than that of the wall of the fenestrae, the endothelial fenestrae hydraulic permeability was calculated by also assuming that the fluid velocity (**v**_*en*_) was related to the local pressure through Darcy’s Law as indicated in Eq. (5) but with the Darcy permeability being *κ*_*en*_, the Darcy permeability of GAGs that fill the endothelial fenestrae. Substituting the above expression for the fluid velocity in the fenestrae into the continuity equation, one also obtains the Laplace equation, Eq. (6), as the governing equation for *P* in the endothelial fenestrae. The local pressure in the mesangium, GBM and the endothelial fenestrae was, therefore, obtained by solving the Laplace equation using finite element method (COMSOL Multiphysics, Stockholm, Sweden). The calculation is presented in Section B of S1 Appendix where the geometry and meshes are shown in Fig B1. At the fenestrae entrance, *P* = *P*_0_, the upstream pressure at the endothelial fenestrae, whereas, at the interfaces between individual layers, the fluid flux must be continuous. To avoid the problem of mesh generation in the finite element scheme (because the meshes had to be very small in order to calculate the fluid velocity through the gap between fibers of the slit diaphragm), the normal velocity at the downstream side of GBM $\left(𝐧\cdot 𝐯{|}_{\begin{array}{c} \;\;\;\;\;\;\;\;\;\;\;\;\;\;\;GBM\\ downstream \end{array}}\right)$ was set to be


$$\begin{array}{c} 𝐧\cdot 𝐯{|}_{\;\;\;\;\;\;\;\;\;\;\;\;\;\;\;\;\begin{array}{c} GBM\\ \;downstream \end{array}}=k_{SD}\left({P|}_{\;\;\;\;\;\;\;\;\;\;\;\;\;\;\;\;\begin{array}{c} GBM\\ \;downstream \end{array}}-P_{f}\right) \end{array}$$ (7)

where *k*_*SD*_ is the hydraulic permeability of the slit diaphragm (of which calculation is discussed in Section A of S1 Appendix). $P{|}_{\begin{array}{c} \;\;\;\;\;\;\;\;\;\;\;\;\;\;\;\;GBM\\ downstream \end{array}}$ is the pressure at the downstream side of GBM, whereas *P*_*f*_ is the downstream hydraulic pressure in Bowman’s space. (The boundary conditions are described in details in Section B of S1 Appendix and shown in Fig B2.)

After obtaining the local pressure at all locations in the mesangial matrix, GBM and the endothelial fenestrae, the fluid velocity in each layer was calculated using Darcy’s Law. The hydraulic permeability of the four-layered barrier (*k*_*four-layered*_) was computed as


$$\begin{array}{c} k_{four-layered}=\frac{\left\langle v_{mesangium}\right\rangle }{P_{\text{0}}-P_{f}} \end{array}$$ (8)

where $\left\langle v_{mesangium}\right\rangle$ was the magnitude of the fluid velocity averaged over the mesangium cross-section. It is worth noting that, because Eq. (6) is linear, the value of *k*_*four-layered*_ is not dependent on the pressure difference; the hydraulic permeability of the four-layered barrier was, then, utilized in the calculation of the transcapillary pressure difference from the reported SNGFR as shown in Eq. (14). The values of the local fluid velocity in the mesangium, GBM and the endothelial fenestrae (**v**_*mesangium*_, **v**_*GBM*_ and **v**_*en*_) computed from the local pressure using Darcy’s law were utilized in the calculation of the sieving coefficient across the four-layered barrier as will be discussed further below.

#### Fluid transport through the shunts at the junction between the filtration surface and the four-layered barrier.

Based on the microscopic image obtained by TEM showing the red blood cells escaping through the voids at the junction between the filtration surface and the four-layered barrier where the shear stress is maximum [28,29], it was assumed in our calculation that the voids or the shunts at the junction between the filtration surface and the four-layered barrier were pores that opened and closed continuously; the pore radius, *R*(*t*), was a periodic function of time, whereas, as shown schematically in Fig 1, the pore length was the GBM thickness (*L*_*GBM*_).

Due to the small Reynolds number $\left(Re=\frac{\rho_{plasma}\left\langle v_{shunt}\right\rangle R_{\text{0}}}{\mu_{plasma}}≅10^{-7}\right)$, the flow passing through the shunts is assumed to be governed by Stokes’ equation and the continuity equation as follows.


$$\begin{array}{c} \nabla P_{shunt}=\mu_{plasma}\nabla^{\text{2}}{𝐯}_{shunt} \end{array}$$ (9a)

$$\begin{array}{c} \nabla \cdot {𝐯}_{shunt}=0 \end{array}$$ (9b)

where *μ*_*plasma*_ is the plasma shear viscosity. *P*_shunt_ and **v**_shunt_ are the pressure and the fluid velocity inside the shunt, respectively. Because of the no-slip boundary condition, the fluid velocity at the pore wall is assumed to be equal to the average velocity in GBM of the filtration surface; $\left\langle v_{GBM}\right\rangle =k_{fs}\left[\Delta P-\Delta \Pi \right]$ where Δ*P* and ΔΠ are the glomerular hydraulic pressure difference and the glomerular osmotic pressure difference, respectively. Due to the linearity of the governing equations, the magnitude of the average fluid velocity of the flow transported through the shunt, $\left\langle v_{shunt}\right\rangle$, can be expressed as


$$\begin{array}{c} \left\langle v_{shunt}\right\rangle =\frac{\Delta \text{\textbackslash{}hspace\{0.17em}}{P}\}\mu_{plasma}f_{T}^{pore}\left(R,L_{GBM}\right)/R+k_{fs}\left[\Delta P-\Delta \Pi \right] \end{array}$$ (10)

where $f_{T}^{pore}\left(R,L_{GBM}\right)$ is the dimensionless flow resistance of a cylindrical pore obtained from a finite element solution of Eqs. (9a) and (9b) that satisfies the no-slip boundary condition at the pore wall (COMSOL Multiphysics, Stockholm, Sweden). For $L_{GBM}\geq 5R$, the difference between $f_{T}^{pore}$ and that of a long cylindrical pore containing a Poiseuille flow was found to be less than 2% [41]; $f_{T}^{pore}$ was, therefore, approximated as the dimensionless flow resistance of the long cylindrical pore $\left(f_{T}^{pore}=8L_{GBM}/R\right)$. It is worth noting than the first term on the right-hand side of Eq. (10) only contains Δ*P* and not Δ*P* - ΔΠ because *R* is much larger than the size of serum albumins that accounts for 80% of the capillary osmotic pressure. The average fluid flow rate through the shunt, $\left\langle R_{\nu }\right\rangle$, can, then, be computed as follows.


$$\begin{array}{c} \left\langle R_{\nu }\right\rangle =\frac{\text{1}}{\tau }\underset{\text{0}}{\overset{\tau }{\int }}N\left(\pi \text{\textbackslash{}hspace\{0.17em}\}R^{\text{2}}\left(t\right)\right)\left\langle v_{shunt}\right\rangle \text{\textbackslash{}hspace\{0.33em}dt\} \end{array}$$ (11a)

where *N* is the average number of shunts per glomerulus and *τ* is the period of the pore opening and closing. Substituting the expression for $\left\langle v_{shunt}\right\rangle$ from Eq. (10) into Eq. (11a), one obtains


$$\begin{array}{c} \left\langle R_{\nu }\right\rangle =\frac{N\text{\textbackslash{}hspace\{0.17em}}{\pi }\text{\textbackslash{}hspace\{0.17em}\}\}\tau \text{\textbackslash{}hspace\{0.33em}\}\underset{\text{0}}{\overset{\tau }{\int }}\text{\textbackslash{}hspace\{0.17em}\left(\frac{R^{\text{4}}\left(t\right)\Delta P}{8\mu_{plasma}L_{GBM}}+k_{\text{\textbackslash{}hspace\{0.17em}}fs\}\text{\textbackslash{}hspace\{0.17em}\}R^{\text{2}}\left(t\right)\left[\Delta P-\Delta \Pi \right]\right)\text{\textbackslash{}hspace\{0.33em}\}dt\} \end{array}$$ (11b)

Two options for *R*(*t*) have been considered. If the shunts open and close periodically and gradually, *R*(*t*) can be described as


$$\begin{array}{c} R\left(t\right)=R_{\text{0}}sin\left(\frac{\pi t}{\tau }\right) \end{array}$$ (12)

where *R*_*0*_ is the maximum shunt radius. If, however, the rupture of GBM occurs suddenly but the closing of the shunt happens gradually, *R*(*t*) can be written as


$$\begin{array}{c} R\left(t\right)=R_{\text{0}}\text{cos}\left(\frac{\pi t}{2\tau }\right) \end{array}$$ (13)

Our calculation, however, demonstrated that the average sieving coefficient calculated using *R*(*t*) as indicated in Eq. (12) only differed slightly from that computed using the expression stated in Eq. (13). The maximum difference between the total sieving coefficient calculated by using Eq. (12) and that computed using Eq. (13) (and employing the parameters of healthy humans stated in Table 1) was 0.15% at *r*_*s*_ = 5.6 nm. Equation (12) was, therefore, employed as the expression for *R*(*t*) in the subsequent calculation [41]. If *L*_*GBM*_ ≤ 4*R*_0_, the average fluid flow rate through the shunt, $\left\langle R_{\nu }\right\rangle$, was calculated using Eq. (11b). If $R_{\text{0}}>L_{GBM}/4$, $\left\langle v_{shunt}\right\rangle$ was calculated using Eq. (10) with $f_{T}^{pore}$ being that of a long cylindrical pore containing a Poiseuille flow in the case that *R*(*t*) ≤$L_{GBM}/4$ where the error was found to be less than 5%. If *R*(*t*) exceeded $L_{GBM}/4$, $\left\langle v_{shunt}\right\rangle$ was calculated as a finite element solution of the Stokes and continuity equations (COMSOL Multiphysics, Stockholm, Sweden) by employing the Lagrangian-quadratic meshes and the default linear solver (UMFPACK); the finite element solution was curvefitted using the polynomial function (MATLAB, Netick, Massachusetts, USA). $\left\langle R_{\nu }\right\rangle$ was, then, obtained using Eq. (11a). An analytical calculation has proved that, if either Eqs. (12) or (13) is employed, the value of $\left\langle R_{\nu }\right\rangle$ does not depend on τ.

#### Calculation of the glomerular hydraulic pressure difference from single nephron glomerular filtration rate (SNGFR).

Based on Eq. (11b), the glomerular hydraulic pressure difference (Δ*P*) can be calculated from SNGFR as shown below.


$$\begin{array}{c} \Delta P=\frac{SNGFR+S_{fs}k_{fs}\Delta \Pi +S_{four-layered}k_{four-layered}\Delta \Pi +N\text{\textbackslash{}hspace\{0.17em}}{\pi }\text{\textbackslash{}hspace\{0.17em}\}\left[\frac{\text{1}}{\text{2}}k_{fs}R_{\text{0}}^{\text{2}}\right]\Delta \Pi \}S_{fs}k_{fs}+S_{four-layered}k_{four-layered}+N\text{\textbackslash{}hspace\{0.17em}\pi \text{\textbackslash{}hspace\{0.17em}\}\left[\frac{\text{3}}{64}\frac{R_{\text{0}}^{\text{4}}}{\mu_{plasma}L_{GBM}}+\frac{\text{1}}{\text{2}}k_{\text{\textbackslash{}hspace\{0.17em}}fs\}\text{\textbackslash{}hspace\{0.17em}\}R_{\text{0}}^{\text{2}}\right]\} \end{array}$$ (14)

Results indicate that the difference between Δ*P* computing by including the effect of $\left\langle R_{\nu }\right\rangle$ and that calculated by excluding such effect is less than 1% if *R*_0_ = 80 nm and *N* ≤ 30 [41]. For other values of *R*_0_, the ratio between $\left\langle R_{\nu }\right\rangle$ and SNGFR is less than 0.005 if *N* is 10 times larger than *N* that yields the best fit to the experimental data. To make the problem more tractable, Δ*P* was estimated from SNGFR by excluding the last term in the nominator and that in the denominator. The employed ΔΠ was the osmotic pressure difference averaged throughout the length of the capillary employing the method introduced by Maddox et al. [42]. It was calculated assuming that the afferent osmotic pressure differences are those of the systemic osmotic pressure [19]. The afferent osmotic pressures differences were 25.5 and 22.6 mmHg for healthy humans and patients with diabetic nephropathy, respectively. The average osmotic pressure difference was 29.5 mmHg for healthy humans and Δ*P* was found to be 36.5 mmHg which is close to the value reported experimentally by Neal et al. [33]. For patients with diabetic nephropathy, the average ΔΠ was 26.4 mmHg and the calculated Δ*P* was higher at 56.3 mmHg. The fluid velocity across the individual components of the glomerular capillary wall can be calculated from the computed Δ*P* and the sieving coefficient of the solutes transported through the filtration surface, the four-layered barrier and the shunts at the junction between the filtration surface and the four-layered barrier can be calculated as will be discussed below.

### Solute transport across the glomerular capillary wall

The total solute sieving coefficient was computed from the sieving coefficient across the filtration surface (*θ*_*filtration surface*_), the sieving coefficient across the four-layered barrier (*θ*_*four-layered*_) and the sieving coefficient through the shunts at the junction between these two barriers averaged over the period that they open and close $\left(\left\langle \theta_{shunt}\right\rangle \right)$. The calculation scheme employed in obtaining these sieving coefficients and the procedure in finding the total sieving coefficient in order to compare with the experimental data from Ficoll sieving coefficients [20,21] are discussed below.

#### Solute filtration across the glomerular filtration surface.

In the calculation of the solute sieving coefficient across the glomerular filtration surface, the ultrastructural model [13,14] was employed where the filtration surface consisted of repeating subunits along the length of the capillary. It has been demonstrated that moderate fluid velocity, small capillary dimension and mixing caused by RBC motion result in the effect of the solute concentration polarization at the upstream end being negligible [3]. The sieving coefficient across the filtration surface (*θ*_*filtration surface*_), the ratio between the downstream and upstream solute concentrations (on either side of the filtration surface), could then be estimated as the product of the solute sieving coefficient across each layer as indicated in Eq. (2). As aforementioned, the calculation of the sieving coefficient across an individual cellular layer was obtained from a solute concentration that was a solution of a steady-state convection-diffusion equation. The solute diffusivity and convection rate were computed by taking into account the solute-fiber interaction. The detailed calculations of *θ*_*en*_, *θ*_*GBM*_ and *θ*_*ep*_ are discussed extensively in Section C of S1 Appendix.

#### Solute transport across the four-layered barrier.

In order to calculate the sieving coefficient across the four-layered barrier, the solute concentration in each individual layer was computed as a steady-state solution of the convection-diffusion equation using finite element method (COMSOL Multiphysics, Stockholm, Sweden). The solute concentration in the mesangial matrix is governed by the steady-state convection-diffusion equation as shown below.


$$\begin{array}{c} \nabla \cdot {𝐍}_{mesangium}=\nabla \cdot \left(-K_{d}^{mes}D_{\infty }\nabla C+K_{c}^{mes}{𝐯}_{mesangium}C\right)=0 \end{array}$$ (15)

where **N**_*mesangium*_ is the solute flux in the mesangium matrix. *C* is the solute concentration. $K_{d}^{mes}$ and $K_{c}^{mes}$ are the solute diffusive and convective hindrance factors, respectively. In our calculation of the hindrance factors, the solutes were seen as being contained in a Brinkman medium. The derivations of $K_{d}^{mes}$ and $K_{c}^{mes}$ are discussed in Section D of S1 Appendix. To determine the solute concentration inside the four-layered barrier, $\theta_{ep}$ must be first calculated as detailed in Section C of S1 Appendix. Then, the solute concentration inside the other three layers was computed by solving the steady-state convection-diffusion equation using finite element scheme (COMSOL Multiphysics, Stockholm, Sweden). The details of the computational procedure of the sieving coefficient across the four-layered including the boundary conditions are presented in Section E of S1 Appendix. In summary, the solute concentration in GBM was first determined, followed by that in the mesangium and that in the endothelial fenestrae. As for the boundary conditions, the downstream solute concentration inside GBM was $C_{B}\Phi_{GBM-fluid}/\theta_{ep}$ where *C*_*B*_ is the solute concentration in Bowman’s space and $\Phi_{GBM-fluid}$ is the partition coefficient at the interface between GBM and the external bulk fluid (the ratio between the solute concentration inside GBM and that in the bulk fluid calculated using Eq. (C15) in S1 Appendix.) The upstream boundary condition for the solute concentration in GBM as well as that in the mesangium and that in the endothelial fenestrae were chosen to ensure that the total solute flux was conserved throughout the transport process across the four-layered barrier. The downstream boundary conditions for solute concentration in the mesangium and that in the endothelial fenestrae followed the approach for solute sieving through multi-layered membranes developed by Boyd and Zydney [43]; the ratio between the solute concentration at the downstream end of the mesangium and that at the upstream end of the GBM was Φ_*mesangium-fluid*_/Φ_*GBM-Fluid*_ where Φ_*mesangium-fluid*_ is the partition coefficient at the mesangium-bulk fluid interface. Similarly, the ratio between the solute concentration at the downstream end of the endothelial fenestrae and that at the upstream end of the mesangium was Φ_*en-Fluid*_/Φ_*mesangium-Fluid*_ where Φ_*en-Fluid*_ is the solute partition coefficient at the endothelial fenestrae-external fluid interface. (The calculation of the partition coefficients are discussed in S1 Appendix.) After *C* was obtained as the finite element solution, the solute sieving coefficient across the four-layered barrier was computed as


$$\begin{array}{c} \theta_{four-layered}=\frac{\left\langle \frac{C_{B}}{C_{\text{0}}}v_{en}\right\rangle }{\left\langle v_{en}\right\rangle }=\frac{\left\langle \frac{C_{B}\Phi_{en-Fluid}}{C_{en}^{inlet}}v_{en}\right\rangle }{\left\langle v_{en}\right\rangle } \end{array}$$ (16)

where *C*_0_ is the upstream solute concentration in the lumen that is equal to $C_{en}^{inlet}/\Phi_{en-Fluid}$ with $C_{en}^{inlet}$ being the concentration right inside the endothelial fenestrae (at the fenestrae inlet). *v*_*en*_ is the magnitude of the fluid velocity in the endothelial fenestrae. The obtained sieving coefficient through the four-layered barrier was, then, combined with the sieving coefficient across the filtration surface and that through the shunt at the junction between the filtration surface and the four-layered barrier to determine the total sieving coefficient. The calculation of the sieving coefficient through the shunts is discussed further below.

#### Solute transport through the shunts at the junction between the filtration surface and the four-layered barrier.

To examine the effect on glomerular size-selectivity of the fluid flow through the shear-induced openings at the junction where the filtration surface meets the four-layered barrier, a mathematical model employing hindered transport theory was developed. As the solute radii (*r*_*s*_) were much smaller than *R*_0_, it was assumed that the hydrodynamic drag on a macromolecule passing through the shunt (**F**_*shunt*_) could be estimated using Faxen’s second law as follows [36].


$$\begin{array}{c} {𝐅}_{shunt}=-6\text{\textbackslash{}hspace\{0.17em}\}\pi \text{\textbackslash{}hspace\{0.17em}\}\mu_{plasma}r_{s}\text{\textbackslash{}hspace\{0.17em}\}\left[\left({𝐔}_{shunt}-{𝐯}_{shunt}\right)-\frac{{r_{\text{\textbackslash{}hspace\{0.17em}}s}^{\text{\textbackslash{}hspace\{0.17em}}}{2}\}\}\text{6}\left(\nabla^{\text{2}}{𝐯}_{shunt}\right)\right] \end{array}$$ (17)

where **U**_*shunt*_ and **v**_*shunt*_ are the particle velocity and the fluid velocity in the shunt, respectively. Balancing the chemical gradient potential and the hydrodynamic drag exerted on the particle and averaging the flux over the cross-section of the shunt, one obtains the following expression for the axial component of the solute flux in the shunt, $\left\langle N_{shunt}\right\rangle$.


$$\begin{array}{c} \left\langle N_{shunt}\right\rangle =-D_{\infty }\frac{d\left\langle C_{shunt}\right\rangle }{dy}+\left\langle v_{shunt}\right\rangle \text{\textbackslash{}hspace\{0.17em}\}\left\langle C_{shunt}\right\rangle +\frac{{r_{\text{\textbackslash{}hspace\{0.17em}}s}^{\text{\textbackslash{}hspace\{0.17em}}}{2}\}\}\text{6}\left(\nabla^{\text{2}}{𝐯}_{shunt}\right)\left\langle C_{shunt}\right\rangle \end{array}$$ (18)

where <*C*_*shunt*_> is the solute concentration in the shunt averaged across the shunt cross-section. $\left\langle v_{shunt}\right\rangle ,$ the magnitude of the average velocity of the fluid flow through the shunt, is computed using Eq. (10). The detailed calculation of the sieving coefficient through the shunts is presented in Section F of S1 Appendix. The solute sieving coefficient (*θ*_*shun*t_) through a shunt of radius *R* is found to be


$$\begin{array}{c} \theta_{shunt}=\frac{\left(K_{c}^{shunt}<v_{shunt}>-A_{shunt}\right)/<v_{shunt}>}{1-\left(1-\left(\frac{K_{c}^{shunt}<v_{shunt}>-A_{shunt}}{<v_{shunt}>}\right)\right)e^{-Pe_{shunt}}} \end{array}$$ (19)

where *Pe*_*shunt*_, $K_{c}^{shunt}$ and *A*_*shunt*_ are defined as shown below.


$$\begin{array}{c} Pe_{shunt}=\frac{\text{\textbackslash{}hspace\{0.17em}}{\left(K_{c}^{shunt}\left\langle v_{shunt}\right\rangle -A_{shunt}\right)}L_{GBM}\}\text{\textbackslash{}hspace\{0.17em}D_{\infty }\} \end{array}$$ (20a)

$$\begin{array}{c} K_{c}^{shunt}=1-\left(\frac{\text{4}}{\text{3}}\right)\frac{{r_{\text{\textbackslash{}hspace\{0.17em}}s}^{\text{\textbackslash{}hspace\{0.17em}}}{2}\}\}R^{\text{2}}+8\mu_{plasma}L_{GBM}k_{fs} \end{array}$$ (20b)

$$\begin{array}{c} A_{shunt}=\left(\frac{\text{4}}{\text{3}}\right)\frac{{k_{fs}r_{\text{\textbackslash{}hspace\{0.17em}}s}^{\text{\textbackslash{}hspace\{0.17em}}}{2}\}\Delta \Pi \}R^{\text{2}}+8\mu_{plasma}L_{GBM}k_{fs} \end{array}$$ (20c)

As aforementioned, *R*(*t*) is a periodic function of time. The sieving coefficient was averaged with the fraction of the fluid volume flow rate through the shunt with the radius of *R*(*t*) as the weighting factor (in a similar way to the way $\theta_{ep}$ was computed) as shown below.


$$\begin{array}{c} \left\langle \theta_{shunt}\right\rangle =\frac{\frac{\text{1}}{\tau }\text{\textbackslash{}hspace\{0.17em}\;}{\underset{\text{0}}{\overset{\tau }{\int }}\text{\textbackslash{}hspace\{0.17em}}N\pi R^{\text{2}}\left(t\right)\left\langle v_{shunt}\right\rangle \text{\textbackslash{}hspace\{0.17em}\}\theta_{shunt}\text{(}t\text{)\textbackslash{}hspace\{0.33em}\}dt\}\}\left\langle R_{v}\right\rangle \end{array}$$ (21)

where $\left\langle \theta_{shunt}\right\rangle$ is the averaged sieving coefficient through the shunt, and $\left\langle R_{\nu }\right\rangle$ is the average fluid flow rate transported through the shunt calculated using Eq. (11b). Similarly to $\left\langle R_{\nu }\right\rangle$, an analytical calculation has proven that, if Eq. (12) or Eq. (13) is employed as an expression for *R*(*t*), the value of $\left\langle \theta_{shunt}\right\rangle$ does not depend on τ. After $\left\langle \theta_{shunt}\right\rangle$ has been determined, the total sieving coefficient based on the combination of the solu*t*e flux across the filtration surface, that across the four-layered barrier, and that through the shunts at the junction between the two barriers, $\left\langle \theta_{total}\right\rangle$, was computed using Eq. (4) with the details being discussed below in the next section.

### Calculation of the total solute sieving coefficient

After the solute sieving coefficients across different components of the glomerular barrier had been computed, it was possible to compute the solute sieving coefficient and compare it to the sieving coefficient of Ficolls from *in vivo* studies [20,21]. If the effects of the presence of the shunts at the junction between the filtration surface and the four-layered barrier was not included, the sieving coefficient (averaged from the solute flux across the filtration surface and that across the four-layered barrier) could be calculated using Eq. (1) with the employed parameters listed in Table 1.

**Fig 13:**
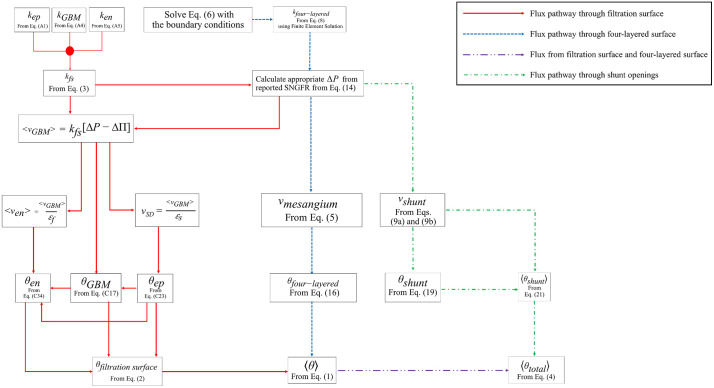
Diagram displaying the computation procedure employed in the calculation.

If the effect of the shear-induced shunts at the junction between the filtration surface and the four-layered on glomerular solute filtration was included, the total sieving coefficient, $\left\langle \theta_{total}\right\rangle$, was computed using Eq. (4) also by using the parameters given in Table 1. The calculation procedure is summarized in a diagram shown in Fig 13. The sensitivity of *θ*_*filtration surface*_ to parameters such as the GBM thickness, the pressure difference across the glomerular barrier, the osmotic pressure difference and the GAG volume fraction in the endothelial fenestrae were examined [19]. It is worth noting that, whereas *θ*_*filtration surface*_ and *θ*_*shunt*_ were obtained as analytical solutions, *θ*_*four-layered*_ was obtained as a finite element solution (COMSOL Multiphysics, Stockholm, Sweden). An analytical calculation has proven that, if either Eq. (12) or Eq. (13) is employed as an expression for *R*(*t*), $\left\langle R_{\nu }\right\rangle ,$$\left\langle \theta_{shunt}\right\rangle$ and, as a result, $\left\langle \theta_{total}\right\rangle$ do not depend on the shunt opening and closing period $\begin{array}{c} \left(\tau \right) \end{array}$.

## Supporting information

S1 Appendix Additional details of mathematical method. (PDF)
