# Supplementary material for: Pathways for glomerular macromolecule filtration: A mathematical model for transport across glomerular filtration surface, mesangium and shear-induced shunts
Source: PLoS Comput Biol. 2026 Jul 20;22(7):e1014503. doi: 10.1371/journal.pcbi.1014503 (PMC13399516; doi:10.1371/journal.pcbi.1014503)
Supplement: S1 Appendix — (PDF) [file pcbi.1014503.s001.pdf]

Supporting Information: S1 Appendix

Additional Details of Mathematical Method

for

Pathways for Glomerular Macromolecule Filtration: A Mathematical Model for Transport across Glomerular Filtration Surface, Mesangium and Shear-Induced Shunts

Numpong Punyaratabandhu, Thapakorn Pankoh, Yuttana Roongthumskul,
Panadda Dechadilok* and Pisut Katavetin*

*Corresponding author. E-mail address: panadda.D@chula.ac.th
*Corresponding author. E-mail address: pkatavetin@yahoo.com

A: Calculation of the hydraulic permeability of the individual cellular layer of the glomerular filtration surface

In the present study, the fluid transport is assumed to be completely extracellular. The hydraulic permeability across the epithelial cell layer, k_{ep}, can be calculated as

$$k_{ep} = \varepsilon_s k_{SD} \qquad \text{(A1)}$$

where ε_s is the fraction of GBM surface not covered by podocytes. k_{SD} is the hydraulic permeability of the slit diaphragm: the average fluid flux filtrated through a row of parallel cylinders with non-uniform spacing $\left(\langle v_{slit}\rangle\right)$ per unit pressure difference as follows (Drumond and Deen, 1995).

$$k_{SD} = \frac{< v_{slit} >}{(\Delta P)_{SD}} = \frac{\int_0^\infty (u+r_c)^2 \; f_T^{-1}(u) g(u) du}{\mu \int_0^\infty (u+r_c)\, g(u) du} \qquad \text{(A2)}$$

where $(\Delta P)_{SD}$ is the pressure drop across the epithelial cell layer and u is the half-width between adjacent cylinders. $g(u)$ is the distribution function of u; $g(u)du$ is the probability of the gap half-width between fibers being in the range of u and u+du. As will be discussed further in Section C, $g(u)$ is assumed to follow a lognormal distribution with the average value and the standard

deviation of u being those reported by Rice et al. (2013). f_T is, on the other hand, the dimensionless flow resistance defined as

$$f_T = \frac{(\Delta P)_{SD}}{\mu v_{slit} / (u + r_c)}. \tag{A3}$$

where μ is the fluid shear viscosity. f_T is calculated as an addition of the dimensionless flow resistance of a row of hindering cylinders (f_R) and the analytically obtained dimensionless flow resistance of a slit channel consisting of two parallel plates (f_P) corresponding to the dimensionless flow resistance of the gap between podocytes that the fluid flow through before reaching the slit diaphragm (Drumond and Deen, 1995; Punyaratabandhu et al., 2017). f_R, the dimensionless flow resistance of a row of parallel cylinders, is obtained from a finite element solution of Stokes' equation (COMSOL, Multiphysics, Stockholm, Sweden); it is compared and found to agree well with values computed by Sangani and Acrivos (1982) and well approximated by Tsay and Weinbaum (1991). Using the parameters stated in Table 1 for healthy humans, k_{SD} is found to be 1,232 nm/s/Pa and, subsequently, k_{ep} is 106 nm/s/Pa.

The hydraulic permeability of an intact GBM (with its surfaces partially covered by the endothelial cells and the epithelial podocytes) has been calculated from the solution of the continuity equation by Drumond and Deen (1994) as shown below.

$$k_{GBM} = \frac{\kappa_{GBM}}{\mu L_{GBM}} \left[1 + \frac{W}{\pi L_{GBM}} \left\{ \frac{1}{n_f} \left[\frac{3}{2} - \ln\left(2\pi\varepsilon_f\right) \right] + \frac{3}{2} - \ln(2\pi\varepsilon_s) \right\} \right]^{-1} \tag{A4}$$

where W is the width of the subunit forming the filtration surface. ε_f is the fraction of GBM upstream surface not covered by the endothelial cells, and ε_s is the fraction of GBM downstream surface not covered by the podocytes. L_{GBM} is the GBM thickness, whereas n_f is the number of endothelial fenestrae per subunit. The above expression was employed in our GBM hydraulic permeability calculation with κ_{GBM}, the Darcy permeability of GBM, computed using Eqs. (C11) - (C13) in Section C. If the physiological parameters and hemodynamic factors are as indicated in Table 1, k_{GBM} is found to be 3.99 nm/s/Pa for healthy humans.

As for the endothelial fenestrae, if the fluid transport is completely extracellular and GAGs filling the fenestrae form the main restricting barrier, k_{en} is computed as follows (Punyaratabandhu et al., 2017).

$$k_{en} = \varepsilon_f k_f = \varepsilon_f \frac{\kappa_{en}}{\mu L_f} \tag{A5}$$

where, as aforementioned, ε_f is the fraction of GBM upstream surface not covered by the endothelial cells, and k_f is the fenestrae hydraulic permeability. μ is the shear fluid viscosity and L_f is the fenestrae length, whereas κ_{en} is the Darcy permeability of the fibrous material filling the fenestrae. As discussed further in Section C, κ_{en} is dependent on the GAG volume fraction in the fenestrae ($\phi_{GAG,en}$).

B: Calculation of the hydraulic permeability of the four-layered barrier

Because the four-layered barrier forms a part of the glomerular capillary wall, its contribution to the overall fluid glomerular filtration is investigated. As discussed in the article, Eq. (6) is employed in the computation of the local pressure in the mesangium matrix, GBM and the endothelial fenestrae; their solutions are obtained using finite element method. Drumond and Deen (1994) demonstrated that a mathematical model employing the two-dimensional geometry with the circular fenestrae replaced by rectilinear openings with the same fractional area was a good approximation that yield the hydraulic permeability close to the three-dimensional geometry. In the present study, this approximation was employed, and the generated mesh is shown in Fig. B1 where the linear dimensions of the four-layered barrier are also stated. The generated meshes were the default triangular meshes. (The number of mesh points is 488688 mesh points). The linear UMFPACK solver was employed. The drop tolerance was set at 10^{-6}; the convergence criteria was that the further mesh refinement led to a change in the hydraulic permeability that was less than 1%.

The boundary conditions required in solving the Laplace equation are shown in fig. B2. At the fenestrae entrance, $P = P_0$, the upstream pressure at the endothelial fenestrae. To avoid the problem of mesh generation in the finite element scheme (as the meshes had to be small enough to calculate the fluid velocity through the slit diaphragm with the fiber diameter and the gap

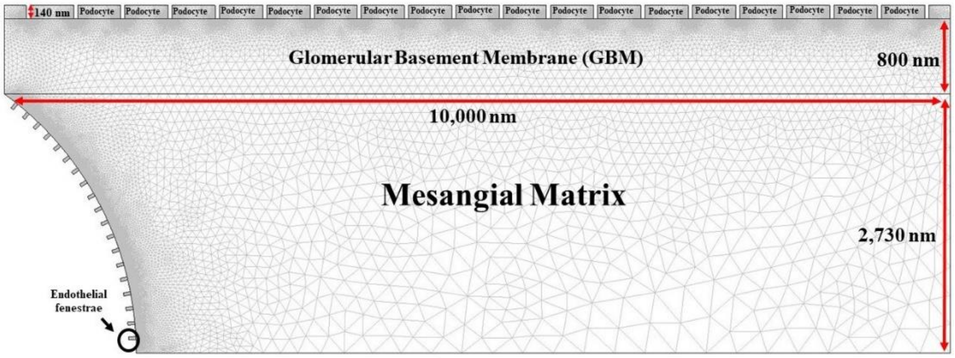

Figure B1: Meshes generated in order to solve the Laplace equation, Eq. (6), to determine the local pressure (*P*) in the case of healthy humans. The above geometry corresponds to a sketch of the four-layered barrier shown in Fig. 1. The length of the interface between GBM and the Mesangial Matrix is 10 µm similarly to the value employed by Hunt et al. (2015). GBM thickness is 400 nm (Deen et al., 2001). The diameter of the glomerular capillary is 6.76 nm (Ellis et al., 1989).

width between fibers much smaller than W, L_{GBM} and the thickness of mesangium), the contribution of the slit diaphragm to fluid restriction was included in the boundary condition at the downstream surface of GBM;

$$\mathbf{n}\cdot\mathbf{v}\Big|_{\substack{GBM \\ downstream}} = -\mathbf{n}\cdot\left(\frac{\kappa_{GBM}}{\mu}\right)\nabla P\Bigg|_{\substack{GBM \\ downstream}} = k_{SD}\left(P\Big|_{\substack{GBM \\ downstream}} - P_f\right) \qquad \text{(B1)}$$

where k_{SD} is the hydraulic permeability of the slit diaphragm (the ratio between the average fluid flux and the pressure difference across the slit diaphragm) as expressed in in Eq. (A2) and **n** is the normal vector pointing outwards from the GBM surface. $\mathbf{v}\Big|_{\substack{GBM \\ downstream}}$ is the fluid velocity at the downstream GBM surface. The downstream boundary condition specified in Eq. (B1) reflects the fact that the normal fluid flux is continuous; the normal fluid flux at the downstream side of GBM equals to the normal fluid flux across the slit diaphragm that is dependent on the difference between $P\Big|_{\substack{GBM \\ downstream}}$,the pressure at the downstream side of GBM, and P_f, the downstream hydraulic pressure in Bowman's space. At the interface between layers, the fluid flux must also

be continuous: $\left(\mathbf{n}\cdot\mathbf{v}_{en}\right)=\left(\mathbf{n}\cdot\mathbf{v}_{mesangium}\right)$ at the upstream mesangial layer surface adjacent to the endothelial fenestrae. At the interface between the mesangial matrix and GBM, $\left(\mathbf{n}\cdot\mathbf{v}_{mesangium}\right)=\left(\mathbf{n}\cdot\mathbf{v}_{GBM}\right)$ with $\mathbf{v}_{mesangium}$, $\mathbf{v}_{GBM}$ and $\mathbf{v}_{en}$ being the fluid velocity in the mesangium, that in GBM and that in the endothelial fenestrae, respectively; all of them were calculated using Darcy's law. At the mesangial matrix surface covered by the endothelial cells, $\mathbf{n}\cdot\mathbf{v}_{mesangium}=0$ as the fluid is assumed to not be able to penetrate into the endothelial cells. For a similar reason, $\mathbf{n}\cdot\mathbf{v}_{en}=0$ at the surface of the endothelial fenestrae, whereas, at the GBM downstream surfaces covered by the podocytes, $\mathbf{n}\cdot\mathbf{v}_{GBM}=0$ as the fluid is assumed not to enter the podocytes.

After obtaining the local pressure in all locations in the mesangial matrix, GBM and the endothelial fenestrae, the fluid velocity in each layer is calculated using Darcy's Law. The hydraulic permeability of the four-layered barrier ($k_{four\text{-}layered}$) was computed using Eq. (8) and utilized in calculating the transcapillary hydraulic pressure difference (ΔP) from SNGFR as mentioned in the article.

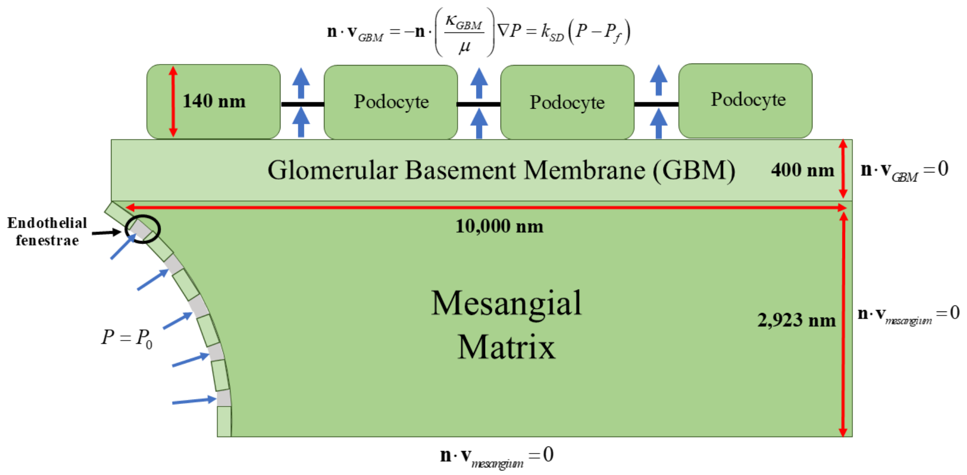

Figure B2: Schematic drawing of the four-layered barrier that includes the glomerular mesangial matrix. Employed boundary conditions for solving the Laplace equation using the finite element method are also specified.

C: Calculation of the solute sieving coefficient across the individual layer of the glomerular filtration surface

Diffusion and convection of test solutes across GBM

In the present study, GBM is assumed to be an isotropic fibrous medium with bimodal fiber structure. As aforementioned, its integrity is believed to be maintained by a network of type IV collagens. Other presented fibers are fibronectin, laminin, entactin, and heparan sulfate proteoglycans with GAG chains attached to core proteins. Deen et al. (2001) demonstrate that a 1:1 mixture of coarse fibers the same size as that of type IV collagens and finer fibers the same size as that of GAGs results in the correct values for the GBM Darcy permeability (κ_{GBM}) if the total fiber volume fraction is 10% and the fluid volume fraction is 90%. Therefore, in the present work, GBM is assumed to contain fluid and two types of fibers: coarser fibers with the diameter being that of a type IV collagen, and finer fibers with the diameter being that of GAG. The volume fraction of each type of fibers is assumed to be 0.05. The concentration of the filtrated solutes can be computed as the solution of a steady-state convection-diffusion equation as follows.

$$\nabla \cdot \mathbf{N}_{GBM} = \nabla \cdot \left(-K_d D_\infty \nabla C + K_c \mathbf{v}_{GBM} C \right) = 0 \quad \text{(C1)}$$

where C is the local solute concentration. $\mathbf{N}_{GBM}$ is the solute flux, whereas D_∞ is the diffusivity of the unconfined solute and $\mathbf{v}_{GBM}$ is the local fluid velocity. K_d is the diffusive hindrance factor (the ratio between the solute diffusivity in GBM and D_∞) whereas K_c is the convective hindrance factor (the ratio between the "apparent" particle velocity during convection and the local fluid velocity).

The contribution of type IV collagens and GAGs to hindered diffusion and convection of solutes in GBM was estimated from an existing hindered transport theory. For Brownian particles confined in a fibrous medium, Brady (1994) proposed that the diffusive hindrance factor could be expressed as a product of F^{-1}, the factor due to the increased hydrodynamic drag caused by the fiber presence, and S, the factor due to particle-fiber steric interaction:

$$K_d = \frac{D_{GBM}}{D_\infty} = F^{-1} S. \quad \text{(C2)}$$

F is the enhanced drag: the ratio between the drag exerted on the confined spherical solute and Stokes' drag on the same solute in an unbounded fluid. Clague and Phillips (1996) computed the enhanced drag on a particle confined in a random array of cylinders of uniform sizes (F_{unif}) and obtained an expression from an ensemble average over many fiber configurations as (Phillips, 2000)

$$F_{unif}(r_s, r_f, \phi) = \exp(A\phi^B) \quad \text{(C3)}$$

where the constants A and B are

$$A = 3.727 - 2.460\left(r_f / r_s\right) + 0.822\left(r_f / r_s\right)^2. \quad \text{(C4)}$$

$$B = 0.358 + 0.366\left(r_f / r_s\right) - 0.0939\left(r_f / r_s\right)^2 \quad \text{(C5)}$$

where ϕ is the fiber volume fraction in the fibrous medium. r_s and r_f are the sphere and fiber radius, respectively. Because GBM was viewed as a fibrous medium containing fibers of two different sizes, Punyaratabandhu et al. (2017) proposed that F was calculated by using an approximation analogous to the approximation proposed by Oseen (1927) and previously employed in estimating the drag on a translating sphere confined between two parallel plates from the drag on a sphere translating near a single plate. Based on Oseen's approximation, the drag on the confined particle can be estimated as a linear superposition: an addition of Stokes'drag on a sphere in an unbounded fluid and an increase in the drag due to solute-GAG interaction as well as an increase due to solute-collagen interaction. F was, therefore, calculated as

$$F \cong 1 + \left(F_{GAG} - 1\right) + \left(F_{collagen} - 1\right) \quad \text{(C6)}$$

where F_{GAG} and $F_{collagen}$ can be calculated using Eqs. (C3) – (C5) as

$$F_{GAG} = F_{unif}\left(r_s, r_f^{GAG}, \phi_{GAG}\right). \quad \text{(C7)}$$

$$F_{collagen} = F_{unif}\left(r_s, r_f^{collagen}, \phi_{collagen}\right). \quad \text{(C8)}$$

where $r_f^{collagen}$ and r_f^{GAG} are the radius of type IV collagens and that of GAGs, respectively. $r_f^{collagen} =$ 3.5 nm, whereas $r_f^{GAG} =$ 0.5 nm. $\phi_{collagen}$ and ϕ_{GAG} are the volume fraction of collagens and GAGs in GBM; in our calculation, $\phi_{collagen} = \phi_{GAG} = 0.05$.

As indicated in Eq. (C2), in addition to the hydrodynamic factor affecting hindered diffusion (F), the steric factor (S), the effects of the solute-fiber steric interaction between solutes and fibers, has to be calculated in order to determine K_d. Due to GAG flexibility causing the effect of the solute-GAG steric interaction likely to be negligible, in the present study, S was computed by including only the solute-collagen steric interaction. It was calculated from the expression proposed by Johnson et al. (1996) for a sphere diffusing in a fibrous medium as

$$S = \exp\left(-0.84\left[\left(1+\frac{r_s}{r_f^{collagen}}\right)^2 \phi_{collagen}\right]^{1.09}\right). \tag{C9}$$

The diffusive hindrance factor (K_d) was computed as a product of S and F^{-1} with S and F being computed using Eqs. (C6) and (C9). As demonstrated by Punyaratabandhu et al. (2017), K_d computed in such way yielded the diffusive permeability (the product of K_d and the equilibrium partition coefficient) that agreed well with the value obtained from an empirical formula of Deen et al. (2001).

In addition to obtaining the expression for the diffusive hindrance factor, the convective hindrance factor (K_c) must also be determined prior to solving the convection-diffusion equation, Eq. (C1). As proposed by Punyaratabandhu et al. (2017), K_c, the ratio between the velocity of the freely suspending solute and that of the local fluid velocity, was calculated from the velocity of the freely suspending particle confined in a Brinkman medium with the Darcy permeability equaling κ_{GBM}. It was computed as the ratio between the drag on a stationary sphere immersed in a uniform flow in a Brinkman medium (divided by Stokes' drag) and the drag on a sphere translating at a constant velocity in a Brinkman medium (that was also similarly scaled) as shown below.

$$K_c = \frac{\left[1+\left(r_s/\sqrt{\kappa_{GBM}}\right)+\frac{\left(r_s/\sqrt{\kappa_{GBM}}\right)^2}{3}\right]}{\left[1+\left(r_s/\sqrt{\kappa_{GBM}}\right)+\frac{\left(r_s/\sqrt{\kappa_{GBM}}\right)^2}{9}\right]}. \tag{C10}$$

where the GBM Darcy permeability (κ_{GBM}) was computed by employing the "volume-weighted resistivity" approach, the mixing rule proposed by Clague and Phillips (1997) for calculating the Darcy permeability of a fibrous medium containing fibers with differing radii from the contributions of each type of fibers as shown below.

$$\frac{1}{\kappa_{GBM}} = \frac{\phi_{GAG}}{\phi_{total}\kappa_{GAG}} + \frac{\phi_{collagen}}{\phi_{total}\kappa_{collagen}} \tag{C11}$$

where ϕ_{total} is the total fiber volume fraction in GBM. ($\phi_{total} = \phi_{GAG} + \phi_{collagen}$.) κ_{GAG} and $\kappa_{collagen}$ are computed using the expression obtained by Amsden (1998) for the Darcy permeability of a fibrous medium as

$$\kappa_{GAG} = 0.31\left(r_f^{GAG}\right)^2\left(\phi_{total}\right)^{-1.17} \tag{C12}$$

$$\kappa_{collagen} = 0.31\left(r_f^{collagen}\right)^2\left(\phi_{total}\right)^{-1.17} \tag{C13}$$

Mattern and Deen (2008), through an examination of Darcy permeabilities of 64 cases of fibrous media containing fibers of multiple sizes, have shown that using the volume weighted resistivity approach in computing the Darcy permeability yielded the Darcy permeability that agreed well with values from experiments and the Darcy permeability calculated using Eq. (C11) was equally applicable for fibrous media containing charged fibers and those containing electrically neutral fibers. κ_{GBM} calculated using Eqs. (C11) - (C13) was found to be 2.2 nm^2; it is within the range of the value reported from experiments at 1.5 – 3 nm^2 (Deen et al., 2001). The convective hindrance factor in GBM is, therefore, calculated by using expressions in Eqs. (C10) – C(13).

After K_d and K_c were determined, Eq. (C1) could be solved by employing the following boundary conditions.

$$C_{upstream} = \Phi_{GBM-en} C_{GBM-en} \quad \text{(C14a)}$$

$$C_{downstream} = \Phi_{GBM-Fluid} C_{GBM-ep} \quad \text{(C14b)}$$

where $C_{GBM\text{-}en}$ is the solute concentration at the GBM-endothelial cell layer interface (right outside GBM) and $C_{GBM\text{-}ep}$ is the solute concentration at the GBM-epithelial cell layer interface (right outside GBM), respectively. $\Phi_{GBM\text{-}fluid}$ is the equilibrium partition coefficient at the GBM-epithelial cell layer interface: the ratio between the downstream solute concentration in GBM and that in the external bulk fluid adjacent to the GBM downstream surface. It is computed by employing the Ogston equation for dilute solution at the interface between a fibrous medium with bimodal fiber structure and a bulk fluid as (Ogston, 1958)

$$\Phi_{GBM-fluid} = \exp\left[-\phi_{collagen}\left(1+\frac{r_s}{r_f^{collagen}}\right)^2 - \phi_{GAG}\left(1+\frac{r_s}{r_f^{GAG}}\right)^2\right]. \quad \text{(C15)}$$

Likewise, $\Phi_{GBM\text{-}en}$ is the equilibrium partition coefficient at the GBM-endothelial fenestrae interface, the ratio between the upstream solute concentration in GBM and that in the endothelial fenestrae adjacent to GBM surface. Because GBM is assumed to contain fibers of two different sizes (those of collagens and GAGs) whereas the endothelial fenestrae are assumed to be filled with uniform-sized fibers (with the fiber diameter being that of GAG), $\Phi_{GBM\text{-}en}$ was estimated based on the Ogston equation as

$$\Phi_{GBM-en} = \frac{\exp\left[-\phi_{collagen}\left(1+\frac{r_s}{r_f^{collegen}}\right)^2 - \phi_{GAG}\left(1+\frac{r_s}{r_f^{GAG}}\right)^2\right]}{\exp\left[-\phi_{GAG,en}\left(1+\frac{r_s}{r_f^{GAG}}\right)^2\right]} \quad \text{(C16)}$$

where, as aforementioned, $\phi_{collagen}$ and ϕ_{GAG} are the volume fraction of collagens and GAGs in GBM. ($\phi_{collagen} = \phi_{GAG} = 0.05$.) $\phi_{GAG,en}$, on the other hand, is the GAG volume fraction in the endothelial fenestrae. Because the average solute flux is continuous throughout all cellular layers and its magnitude is equal to $C_B V_B$ where C_B and V_B are the solute concentration and the magnitude of fluid velocity in Bowman's space, respectively, the steady-state solution of Eq. (C1) yields the following expression for the solute sieving coefficient across GBM.

$$\theta_{GBM} = \frac{C_{GBM-ep}}{C_{GBM-en}} = \frac{\Phi_{GBM-en} K_c}{\theta_{ep}\left[1-\exp(-Pe_{GBM}^{intact})\right] + \Phi_{GBM-fluid} K_C \exp(-Pe_{GBM}^{intact})} \qquad \text{(C17)}$$

where Pe_{GBM}^{intact} appeared in Eq. (C17) is the Peclet number, the dimensionless parameter indicating the relative contribution of convection and diffusion to solute transport in an intact GBM (with its surfaces partially blocked by the endothelial cells and the podocytes). Edwards et al. (1999) obtained the correlation between Pe_{GBM}^{intact} and Pe_{GBM}^{bare} (the Peclet number characterizing solute transport across a bare GBM without the surface blockage) from the steady-state solution of Eq. (C1) as

$$Pe_{GBM}^{intact} = Pe_{GBM}^{bare}\left[1 + 0.7366\left(1-\varepsilon_f \varepsilon_s\right)^{11.9864}\left(L_{GBM}/W\right)^{-1.2697}\right] \qquad \text{(C18)}$$

where the expression in the bracket on the right-hand side of Eq. (C18) is the effect of the GBM surface being partially blocked by the two cellular layers on the Peclet number of the intact GBM; ε_f and ε_s are the fractions of GBM surfaces not covered by the endothelial cells and the epithelial podocytes, respectively. The Peclet number characterizing solute transport across a bare GBM, Pe_{GBM}^{bare}, can be written as follows.

$$Pe_{GBM}^{bare} = (K_c \left\langle v_{GBM} \right\rangle L_{GBM}) / (K_d D_\infty) \qquad \text{(C19)}$$

where $\left\langle v_{GBM} \right\rangle$ is the magnitude of the average fluid velocity across GBM. An inspection of Eq. (C17) demonstrates that the only parameter remains to be determined prior to obtaining the solute sieving coefficient through GBM (θ_{GBM}) is the sieving coefficient through the epithelial slit (θ_{ep}). This is due to the fact that, for multi-layered membranes, the value of the sieving coefficient across an individual layer is dependent on values of the sieving coefficients across more downstream layers. The calculation of θ_{ep} is presented below.

Solute transport across the epithelial slit

As aforementioned, the epithelial cell layer of the glomerular capillary wall consists of a slit diaphragm connecting the podocytes. Following the available electron micrographs (with the highest resolution) of the epithelial slit structure obtained by Rice et al. (2013), in the present study, the epithelial slit is represented as a row of parallel cylinders. Solving the steady-state convection-diffusion equation for a solute concentration across a row of parallel cylinders of uniform spacing, Drumond and Deen (1995) obtained, to good approximation, an expression for the sieving coefficient of spherical solutes as follows.

$$\theta_{slit}(u) = \frac{1-(r_s/u)}{1-(1-\exp((-Pe_{SD}L_{GBM-slit})/r_c)(1-\exp(-A_{slit}Pe_{SD}))(r_s/u)} \quad \text{(C20)}$$

where r_s and r_c are the solute radius and the cylinder cross-section radius, respectively. u is the half-width of the gap between adjacent cylinders and $L_{GBM\text{-}slit}$ is the distance between the slit diaphragm and the GBM surface. The parameter A appearing in the above equation is found to be

$$A_{slit} = \frac{3.65}{\left(r_c/(u+r_c)\right)} + \frac{0.573}{\left(u/(u+r_c)\right)} \quad \text{(C21)}$$

Also appeared in Eq. (C20) is the Peclet number characterizing the solute transport across the row of cylinder (Pe_{SD}) defined as (Drumond and Deen, 1995)

$$Pe_{SD} = (v_{SD}r_c)/D_\infty \quad \text{(C22)}$$

where v_{SD}, the magnitude of the upstream fluid velocity far from the row of cylinders, is equal to $\langle v_{GBM}\rangle/\varepsilon_s$.

As aforementioned, the size of the gap between fibers of the epithelial slit are found to be non-uniform. Because Bowman's Space is a chamber with the solute flux transported into it being the product of the fluid flux and C_B (the solute concentration in Bowman's Space), Drumond and Deen (1995) proposed that the average sieving coefficient through the slit diaphragm could be computed as

$$\theta_{ep} = \frac{C_{GBM-ep}}{C_B} = \int_0^\infty \theta_{slit}(u)G(u)\,du \quad \text{(C23)}$$

where $G(u)\,\mathrm{d}u$ is the fraction of the fluid volume flow rate through the interfiber spacing with the gap half-width being in the range of u and $u+\mathrm{d}u$, and can be obtained as

$$G(u)du = \frac{\left(u+r_c\right)v_{slit}\left(u\right)g(u)du}{\int_0^\infty (u+r_c)v_{slit}\left(u\right)g(u)du} = \frac{\left(u+r_c\right)^2 f_T^{-1}\left(u\right)g(u)du}{\int_0^\infty (u+r_c)^2 f_T^{-1}\left(u\right)g(u)du} \qquad \text{(C24)}$$

where $g(u)\,du$ is the probability of the gap half-width between fibers being in the range of u and $u+\mathrm{d}u$. v_{slit} is the magnitude of the fluid velocity through a row of parallel cylinders with the gap half-width of the spacing between adjacent fibers being u if the direction of the fluid velocity is perpendicular to the cylinder axes. f_T is the dimensionless flow resistance defined in Eq. (A3)

The distribution function of the half-width of the gap between adjacent fibers, $g(u)$, was determined based on the reported value for the dimension of the gap between fibers from the observation of Rice et al. (2013). Following the approach of Drumond and Deen (1995), the requirement for $g(u)$ is that, as $u \to 0$ or $u \to \infty$, $g(u) \to 0$. Possible choices include the lognormal and gamma distributions; Punyaratabandhu (2015) demonstrated that θ_{ep} calculated by assuming that $g(u)$ followed a lognormal distribution is graphically indistinguishable from that computed by assuming that $g(u)$ followed a gamma distribution if the mean and standard deviation of u were assumed to be the same for both cases. In the present study, $g(u)$ is assumed to follow the lognormal distribution and can be expressed as

$$g(u) = \frac{\exp\left(-(\ln u - \eta)^2 / 2\sigma^2\right)}{u\sigma\sqrt{2\pi}} \qquad \text{(C25)}$$

where σ and η are constant and related to the mean and standard deviation of u as shown below.

$$\langle u \rangle = \exp\left(\eta + \left[\sigma^2/2\right]\right) \qquad \text{(C26)}$$

$$\sqrt{\langle u^2 \rangle} = \left[\left(\exp(\sigma^2)-1\right)\exp(2\eta+\sigma^2)\right]^{1/2} \qquad \text{(C27)}$$

Based on the values reported by Rice et al. (2013), in the present work, $\langle u \rangle$ = 11 nm, and $\sqrt{\langle u^2 \rangle}$ is set at 2 nm, resulting in σ = 0.1803 and η = 2.3816. After $g(u)$ was determined, the sieving

coefficient across the epithelial slit (θ_{ep}) could be computed using the expressions stated in Eqs. (C20) – (C27), and, subsequently, the sieving coefficient across GBM (θ_{GBM}) could be calculated using Eq. (C17). As indicated in Eq. (2), in order to obtain the sieving coefficient across the glomerular filtration surface, the sieving coefficient across the endothelial cell layer must be determined; its calculation is discussed below.

Solute transport through the endothelial fenestrae

Electron microscopic images of the endothelial cell layer of the glomerular barrier have shown that it has a large fenestrated area; these fenestrae are 50-100 nm in diameter and contain fibers believed to be proteoglycans with the core proteins and GAG chains. In the present study, the endothelial fenestrae are viewed as being full of fibers the same size as that of GAGs. The solute concentration in the fenestrae is governed by the convection-diffusion equation but with the fluid velocity replaced by $\mathbf{v}_{en}$, the local fluid velocity in the endothelial fenestrae. The diffusive and convective hindrance factors are K_d^{en} and K_c^{en}. As the size of the fenestrae diameter is much larger than those of Ficolls utilized in experiments conducted by Blouch et al. (1997) and Andersen et al. (2000), the contribution of the particle hydrodynamic and steric interaction with the fenestrae wall is assumed to be negligible, and only the solute interaction with GAGs contributes to the solute hindered diffusion and convection in the endothelial fenestrae. K_d^{en}, the ratio between the solute diffusivity in the fenestrae, is calculated by including the particle-GAG hydrodynamic interaction as shown below.

$$K_d^{en} = F_{unif}^{-1}\left(r_s, r_f^{GAG}, \phi_{GAG,en}\right) \quad \text{(C28)}$$

where $F_{unif}\left(r_s, r_f^{GAG}, \phi_{GAG,en}\right)$ is the enhanced drag calculated using Eqs. (C3) – (C5): the drag exerted on the solute translating in the fibrous medium containing fibers with the same radii as that of GAG (divided by Stokes' drag). *S*, the steric factor as indicated in Eq. (C2), was assumed to be 1 due to GAG flexibility. K_c^{en}, the ratio between the velocity of the freely suspending particle in the endothelial fenestrae and the fluid velocity, on the other hand, was computed from the velocity of the freely suspending particle in the Brinkman medium with the Darcy

permeability being that of the fibrous medium contained in the fenestrae (similarly to the calculation of the convective hindrance factor for solutes in GBM) as follows.

$$K_c^{en} = \frac{\left[1 + \left(r_s / \sqrt{\kappa_{en}}\right) + \frac{\left(r_s / \sqrt{\kappa_{en}}\right)^2}{3}\right]}{\left[1 + \left(r_s / \sqrt{\kappa_{en}}\right) + \frac{\left(r_s / \sqrt{\kappa_{en}}\right)^2}{9}\right]}. \tag{C29}$$

where κ_{en} is the Darcy permeability of the material that fills the fenestrae computed from the expression obtained by Amsden (1998) as

$$\kappa_{en} = 0.31\left(r_f^{GAG}\right)^2 \left(\phi_{GAG,en}\right)^{-1.17} \tag{C30}$$

After K_d^{en} and K_c^{en} are determined, the steady-state convection diffusion equation can be solved by employing the following boundary conditions.

$$C_{upstream}^{en} = \Phi_{en\text{-}fluid} C_0 \tag{C31}$$

$$C_{downstream}^{en} = C_{GBM-en} \tag{C32}$$

where C_0 is the solute concentration in the capillary lumen and $C_{GBM\text{-}en}$ is the solute concentration at the GBM-endothelial cell layer interface (right outside GBM), respectively. $\Phi_{en\text{-}Fluid}$ is the solute equilibrium partition coefficient, the ratio between the solute concentration at the upstream end of the fenestrae and that in the external fluid in the lumen. It was computed using the Ogston equation for dilute solutions as show below.

$$\Phi_{en-Fluid} = \exp\left[-\phi_{GAG,en}\left(1 + \frac{r_s}{r_f^{GAG}}\right)^2\right]. \tag{C33}$$

Because the average solute flux is continuous throughout all cellular layers and its magnitude is equal to $C_B V_B$ where C_B and V_B are the solute concentration and the magnitude of fluid velocity in Bowman's space, respectively, the steady-state solution yields the following expression for the sieving coefficient through the endothelial fenestrae.

$$\theta_{en} = \frac{C_{GBM-en}}{C_0} = \frac{\Phi_{en-Fluid} K_c^{en}}{\theta_{GBM}\theta_{ep}\left(1-\exp(-Pe_{en})\right) + K_c^{en}\exp(-\mathrm{Pe}_{en})} \quad \text{(C34)}$$

where Pe_{en} is the Peclet number characterizing solute transport through the material filling the endothelial fenestrae defined as

$$Pe_{en} = \frac{\langle v_{en} \rangle K_c^{en} L_f}{K_d^{en} D_\infty} \quad \text{(C35)}$$

where $<v_{en}>$ is the magnitude of the average fluid velocity in the fenestrae $\left(\varepsilon_f \langle v_{en} \rangle = \langle v_{GBM} \rangle\right)$ and L_f is the fenestrae length, respectively. The absence of the partition coefficient in the denominator of Eq. (C34) is due to the fact that $C_{GBM\text{-}en}$ is defined as the downstream solute concentration right outside GBM (Punyaratabandhu et al., 2017). It is worth noting that, as demonstrated by the expression in Eq. (C34), the value of sieving coefficient across the endothelial cell layer, θ_{en}, is dependent on the values of the sieving coefficients across the two more downstream layers. After θ_{en}, θ_{GBM} and θ_{ep} are determined, the total sieving coefficient across the filtration surface ($\theta_{filtration\ surface}$) can be computed as their product as shown in Eq. (2).

D: Calculation of the hindrance factors of the solutes in the mesangium matrix

The balance between the chemical potential gradient and the drag on the sphere moving at the velocity **U** in a mesangial matrix with the fluid flowing at the velocity **V** can be written as follows.

$$-kT\nabla \ln C - 6\pi\mu a(K_1\mathbf{U} - K_2\mathbf{v}_{mesangium}) = 0 \quad \text{(D1)}$$

If the mesangial matrix is assumed to be an unbounded Brinkman medium (or a Brinkman with a linear dimension much larger than the size of the sphere), K_1 is the ratio between the drag on a sphere moving in a quiescent Brinkman medium and Stokes' drag. On the other hand, K_2 is the ratio between the drag on a stationary sphere blocking a flow in a Brinkman medium and Stokes' drag. Their expressions are as shown below (Sugihara-seki, 2004).

$$K_1 = \left[1 + \left(r_s / \sqrt{\kappa_{mes}} \right) + \frac{\left(r_s / \sqrt{\kappa_{mes}} \right)^2}{9} \right] \tag{D2}$$

$$K_2 = \left[1 + \left(r_s / \sqrt{\kappa_{mes}} \right) + \frac{\left(r_s / \sqrt{\kappa_{mes}} \right)^2}{3} \right] \tag{D3}$$

Equation (D1) can be rewritten as

$$\begin{aligned} \mathbf{N}_{mesangium} &= C\mathbf{U} = -\left(\frac{kT}{6\pi\mu a K_1} \right) \nabla C + \left(\frac{K_2}{K_1} \right) \mathbf{v}_{mesangium} C \\ &= -D_\infty K_d^{mes} \nabla C + K_c^{mes} \mathbf{v}_{mesangium} C \end{aligned} \tag{D4}$$

where $\mathbf{N}_{mesangium}$ is the solute flux in the mesangium. The diffusive hindrance factor of the solute in the glomerular mesangium, K_d^{mes}, the ratio between the solute diffusivity in the glomerular mesangium, and the diffusivity of the same solute in an unbounded fluid, is

$$K_d^{mes} = K_1^{-1} = \left[1 + \left(r_s / \sqrt{\kappa_{mes}} \right) + \frac{\left(r_s / \sqrt{\kappa_{mes}} \right)^2}{9} \right]^{-1} . \tag{D5}$$

The expression in Eqs. (D4) and (D5) is based on the fact that the diffusivity is the ratio between the thermal energy and the drag coefficient; the increase in the hydrodynamic drag corresponds to the diffusivity reduction. The convective hindrance factor of the solute in the mesangial matrix $\left(K_c^{mes} \right)$ was calculated as

$$K_c^{mes} = \frac{K_2}{K_1} = \frac{\left[1 + \left(r_s / \sqrt{\kappa_{mes}} \right) + \frac{\left(r_s / \sqrt{\kappa_{mes}} \right)^2}{3} \right]}{\left[1 + \left(r_s / \sqrt{\kappa_{mes}} \right) + \frac{\left(r_s / \sqrt{\kappa_{mes}} \right)^2}{9} \right]} . \tag{D6}$$

E: Calculation of the sieving coefficient across the four-layered barrier

In order to calculate the sieving coefficient across the four-layered barrier, the solute concentration in each individual layer was computed as a steady-state solution of the convection diffusion equation using finite element method (COMSOL Multiphysics, Stockholm, Sweden) starting from the solute concentration in GBM followed by that in the mesangium and the endothelial fenestrae, respectively. The solute concentration in GBM is governed by Eq. (C1); the hindrance factors are calculated using Eqs. (C2) – (C10). The governing equation for C in the mesangium matrix is Eq. (15) with K_d^{mes} and K_c^{mes} computed using Eqs. (D5) and (D6), whereas the governing equations for the solute concentration in the endothelial fenestrae is similar to that of C in the endothelial fenestrae in the glomerular filtration surface with the diffusive hindrance factors obtained by employing the expressions in Eqs. (C28) and (C29), respectively. The difference is that $\mathbf{v}_{GBM}$ and $\mathbf{v}_{en}$ are fluid velocities in GBM and the endothelial fenestrae computed using Darcy's law as described in Section B; their magnitudes are different from those of $\mathbf{v}_{GBM}$ and $\mathbf{v}_{en}$ in the glomerular filtration surface. In the finite element scheme, the default triangular meshes and the linear UMFPACK solver were employed. The convergence criteria was that the mesh refinement led to a less than 1% change in the sieving coefficient.

Equation (15) and all the convection-diffusion equations for all cellular layers must be solved by employing appropriate boundary conditions as shown in Fig. E1. First, θ_{ep} must be calculated using Eqs. (C20) – (C27). Following the approach of Edwards et al. (1999), at the GBM downstream surface, the solute concentration is as follows.

$$C\big|_{GBM-ep \text{ interface}} = C_B \Phi_{GBM-Fluid} \big/ \theta_{ep} \qquad \text{(E1)}$$

where C_B is the downstream solute concentration in Bowman's space and $\Phi_{GBM\text{-}Fluid}$ is the solute partition coefficient at the interface between the downstream GBM surface and the bulk fluid computed using Eq. (C15). In addition, we must ensure that the solute flux is continuous; the average normal solute flux at the upstream surface of GBM must be equal to

$N_f = C_B V_B = C_B \left\langle \varepsilon_s k_{SD} \left(P\big|_{\substack{GBM \\ downstream}} - P_f \right) \right\rangle$ where V_B is the magnitude of the fluid velocity in Bowman's space and the product $C_B V_B$ is the magnitude of the downstream flux into Bowman's space, N_f. This results in

$$\mathbf{n}\cdot\mathbf{N}\big|_{\substack{GBM\\upstream}} = C_B\left(\mathbf{n}\cdot\mathbf{v}\big|_{\substack{GBM\\upstream}}\right) \qquad \text{(E2)}$$

where $\mathbf{N}\big|_{\substack{GBM\\upstream}}$ and $\mathbf{v}\big|_{\substack{GBM\\upstream}}$ are the solute flux and the fluid velocity at the upstream GBM surface, respectively. This is because the fluid flux is also continuous;

$$V_B = \left\langle \varepsilon_s k_{SD}\left(P\big|_{\substack{GBM\\downstream}} - P_f\right)\right\rangle = \left\langle \mathbf{n}\cdot\mathbf{v}\big|_{\substack{GBM\\upstream}}\right\rangle \qquad \text{(E3)}$$

where, as aforementioned, ε_s is the fraction of GBM downstream surface not covered by the podocytes. $\left\langle \mathbf{n}\cdot\mathbf{N}\big|_{\substack{GBM\\upstream}}\right\rangle$ is, therefore, equal to N_f, indicating that the solute flux is continuous. At the GBM downstream surface covered by the podocytes, $\mathbf{n}\cdot\mathbf{N}_{GBM} = 0$ as the flow was assumed to be extracellular.

After the solute concentration in GBM was obtained, the solute concentration in the mesangium was computed by solving the steady-state convection-diffusion equation: Eq. (15) in the article. Following the approach for solute sieving through multi-layered membranes developed by Boyd and Zydney (1997), the ratio between the solute concentration at the upstream end of the GBM and that at the downstream end of the mesangium is $\Phi_{GBM\text{-}fluid}/\Phi_{mesangium\text{-}Fluid}$, resulting in the solute concentration at the downstream end of the mesangium, $C\big|_{\substack{mesangium\\downstream}}$, being

$$C\big|_{\substack{mesangium\\downstream}} = \left(\frac{\Phi_{mesangium-fluid}}{\Phi_{GBM-fluid}}\right) C\big|_{\substack{GBM\\upstream}} \qquad \text{(E4)}$$

where $C\big|_{\substack{GBM\\upstream}}$ is the upstream solute concentration inside GBM. $\Phi_{GBM\text{-}fluid}$ is the partition coefficient between GBM and the bulk fluid calculated using Eq. (C15). $\Phi_{mesangium\text{-}fluid}$ is the partition coefficient between the mesangium and the bulk fluid that is computed using Eq. (C30) and Eq. (C33) but with κ_{en} replaced by κ_{mes}.

At the upstream surface of the mesangium, the boundary condition was chosen to ensure that the total flux was conserved similarly to the upstream boundary condition for the solute concentration in GBM;

$$\left(\mathbf{n} \cdot \mathbf{N}\Big|_{\substack{mesangium \\ upstream}} \right) = C_B \left(\mathbf{n} \cdot \mathbf{v}\Big|_{\substack{mesangium \\ upstream}} \right) \qquad \text{(E5)}$$

where $\mathbf{N}\Big|_{\substack{mesangium \\ upstream}}$ and $\mathbf{v}\Big|_{\substack{mesangium \\ upstream}}$ are the upstream mesangial solute flux and the upstream fluid velocity in the mesangium, respectively. This is because $V_B = \varepsilon_f \left\langle \mathbf{n} \cdot \mathbf{v}\Big|_{\substack{mesangium \\ upstream}} \right\rangle$. (The upstream mesangial surface is partially blocked by the endothelial cells with ε_f being the fraction of the surface not covered by the endothelial cells.) Therefore, $\varepsilon_f \left\langle \mathbf{n} \cdot \mathbf{N}\Big|_{\substack{mesangium \\ upstream}} \right\rangle = C_B V_B = N_f$, indicating that the average solute flux was conserved. At the mesangium surface covered by the endothelial cells, $\mathbf{n} \cdot \mathbf{N}_{mesangium} = 0$ as it was assumed that the solute flux did not penetrate the endothelial cells.

After the mesangial solute concentration was calculated, the solute concentration in the innermost layer, the endothelial fenestrae, must be determined by solving the steady-state convection-diffusion equation. As for the boundary conditions, the ratio between the solute concentration at the upstream end of the mesangium and that at the downstream end of the endothelial fenestrae was $\Phi_{mesangium\text{-}Fluid}/\Phi_{en\text{-}Fluid}$ where $\Phi_{en\text{-}Fluid}$ and $\Phi_{mesangium\text{-}Fluid}$ are the solute partition coefficient at the endothelial-bulk fluid interface and that at the mesangium-external fluid interface, respectively (Boyd and Zydney, 1997).

$$C\Big|_{\substack{endothelial \\ downstream}} = \left(\frac{\Phi_{en-fluid}}{\Phi_{mesangium-fluid}} \right) C\Big|_{\substack{mesangium \\ upstream}} \qquad \text{(E6)}$$

where $C\Big|_{\substack{endothelial \\ downstream}}$ is the solute concentration at the downstream end inside the endothelial fenestrae and $C\Big|_{\substack{mesangium \\ upstream}}$ is the upstream solute concentration inside the mesangium.

At the inlet of the endothelial fenestrae, the boundary condition was chosen to ensure that the total solute flux was conserved similarly to the upstream boundary condition for the solute concentration in GBM and that in the mesangial matrix as shown below.

$$\varepsilon_f \left(\mathbf{n} \cdot \mathbf{N}_{en} \big|_{en-lumen \text{ interface}} \right) = \varepsilon_f \left(\mathbf{n} \cdot \mathbf{v}\big|_{\substack{en \\ upstream}} \right) C_B \qquad \text{(E7)}$$

where $\mathbf{N}\big|_{en-lumen\ interface}$ and $\mathbf{v}\big|_{\substack{en \\ upstream}}$ are the solute flux and the fluid velocity at the inlet of the endothelial fenestrae, respectively. Due to the fact that $\varepsilon_f \left\langle \mathbf{n} \cdot \mathbf{v}\big|_{\substack{en \\ upstream}} \right\rangle = V_B$ because of fluid flux continuity, $\varepsilon_f \left\langle \mathbf{n} \cdot \mathbf{N}_{en} \big|_{en-lumen \text{ interface}} \right\rangle = N_f$ and the average solute flux is conserved. In addition, at the wall of the endothelial fenestrae, the solute flux must be zero. After C was obtained as the finite element solution, the solute sieving coefficient across the four-layered barrier can be computed using Eq. (16).

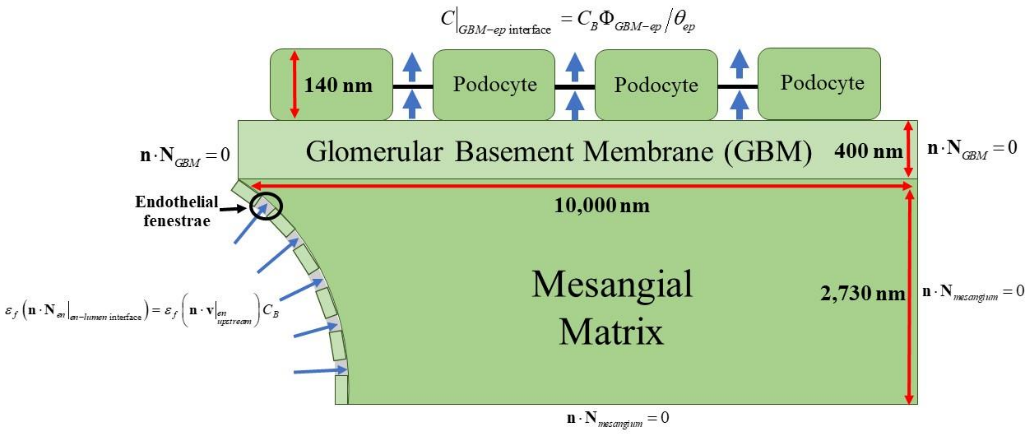

Figure E1: Schematic drawing of the four-layered barrier that includes the glomerular mesangial matrix. Employed boundary conditions for solving the diffusion-convection equation using the finite element method are also specified.

F: Calculation of the sieving coefficient through the shunt at the junction between the glomerular filtration surface and the four-layered barrier

Because of the values of R_0 and L_{GBM} utilized in our calculation, $\mathbf{v}_{shunt}$ is unidirectional at most locations and is well-approximated as the addition of the fluid velocity of the Poiseuille flow and the velocity at the pore wall (with its magnitude being $\langle v_{GBM} \rangle = k_{fs}[\Delta P - \Delta\Pi]$). Based on Eq. (10), the hydraulic pressure difference can be written as

$$\Delta P = \frac{\langle v_{shunt} \rangle + k_{fs}\Delta\Pi}{\left(\frac{R^2}{8\mu_{plasma} L_{GBM}} + k_{fs} \right)} \tag{F1}$$

Substituting the expression in Eq. (F1) into Eq. (9a), one founds that $\nabla^2 \mathbf{v}_{shunt}$ is well-approximated as being constant and is related to $\langle v_{shunt} \rangle$ as shown below.

$$\nabla^2 \mathbf{v}_{shunt} \cong \frac{-8\left(\langle v_{shunt} \rangle + k_{fs}\Delta\Pi\right)}{R^2 + 8\mu_{plasma} L_{GBM} k_{fs}} \tag{F2}$$

A substitution of the expression in Eq. (F2) into Eq. (18) yields

$$\langle N_{shunt} \rangle = -D_\infty \frac{d\langle C_{shunt} \rangle}{dy} + K_c^{shunt} \langle v_{shunt} \rangle \langle C_{shunt} \rangle - A_{shunt} \langle C_{shunt} \rangle \tag{F3}$$

where y is defined as shown in Fig. 1. K_c^{shunt} and A_{shunt} are defined as indicated in Eqs. (20b) and (20c), respectively. An integration of Eq. (F3) yields the following expression.

$$\int_{y_{shunt}^{entrance}}^{y_{shunt}^{entrance}+L_{GBM}} -\frac{1}{D_\infty} dy = \int_{\langle C_{shunt}(y=y_{shunt}^{entrance}) \rangle}^{\langle C_{shunt}(y=y_{shunt}^{entrance}+L_{GBM}) \rangle} \frac{1}{\langle N_{shunt} \rangle - (K_c^{shunt} \langle v_{shunt} \rangle - A_{shunt}) \langle C_{shunt} \rangle} d\langle C_{shunt} \rangle \tag{F4}$$

where $y_{entrance}^{shunt}$ is the value of y at the inlet of the shunt. The expression for the average axial solute flux flowing into a chamber is as follows.

$$\begin{aligned} \langle N_{shunt} \rangle &= C\left(y = y_{shunt}^{entrance} + L_{GBM}\right) \langle v_{shunt} \rangle \\ &= \frac{\left[K_c^{shunt} \langle v_{shunt} \rangle \text{-} A_{shunt}\right] C\left(y = y_{shunt}^{entrance}\right)}{1 - \left(1 - [K_c^{shunt} < v_{shunt} > - A_{shunt}] / < v_{shunt} >\right) e^{-Pe_{shunt}}} \end{aligned} \tag{F5}$$

where Pe_{shunt} is defined in Eq. (20a). The solute sieving coefficient through the shunt or $\theta_{shunt} = C\left(y_{shunt}^{entrance} + L_{GBM}\right) / C\left(y_{shunt}^{entrance}\right)$ can, therefore, be expressed as indicated in Eq. (19) In the present study, if $R(t) \leq L_{GBM}/4$, θ_{shunt} is computed using Eq. (19). If $R(t)$ exceeds $L_{GBM}/4$ (for instance, $R(t)$ > 100 nm for healthy humans), θ_{shunt} is set to equal to 1. If R_0 exceeds 500 nm, $\langle\theta_{shunt}\rangle$ is set to equal to 1. (At R_0 = 500 nm, $\langle\theta_{shunt}\rangle$ is found to be 0.9999997 for healthy humans and 0.9999976 for patients with diabetic nephropathy if r_s = 3.6 nm, for instance.)

G: Numerical scheme allowing R_0 to follow the lognormal distribution

In the numerical method allowing R_0, the maximum shunt radius during the periodic opening and closing of the shunt, to follow the lognormal distribution, R_0 was chosen randomly from the lognormal distribution using the command lognrnd (MATLAB, Netick, Massachusetts, USA) where σ and η are constant and related to the mean and standard deviation (SD) of R_0 through Eqs. (C26) and (C27), respectively. $<R_0>$, the mean value of R_0, was set at 80 nm, 200 nm, 460 nm and 1125 nm, where *N*, the average number of shunts per glomerulus, for each $<R_0>$ being the value best fitting the experimental data (Blouch et al., 1997; Andersen et al., 2000). The process of determining $\langle\theta_{shunt}\rangle$ and $\langle R_V\rangle$ was to randomly choose R_0 from a lognormal distribution which was repeated 100,000 times for each set of *N* and the average value of $\langle\theta_{shunt}\rangle\langle R_V\rangle / SNGFR$ was obtained. The total sieving coefficient was, then, calculated according to Eq. (4).

H: List of symbols

ΔP	transcapillary hydraulic pressure difference
$(\Delta P)_{SD}$	pressure drop across the epithelial cell layer
$\Delta \Pi$	transcapillary osmotic pressure difference
ε_f	fraction of GBM surface not covered by the endothelial cells
ε_s	fraction of GBM surface not covered by podocytes
θ_{en}	solute sieving coefficient through the endothelial fenestrae
θ_{ep}	solute sieving coefficient through the epithelial cell layer
$\theta_{filtration\ surface}$	sieving coefficient across the filtration surface
$\theta_{four\text{-}layered}$	sieving coefficient across the four-layered barrier
θ_{GBM}	solute sieving coefficient through GBM
θ_{shunt}	solute sieving coefficient through the shunts
$\langle \theta \rangle$	average solute sieving coefficient calculated from the solute flux across the filtration surface and the solute flux across the four-layered barrier
$\langle \theta_{shunt} \rangle$	average sieving coefficient through the periodically opening and closing shunts
$\langle \theta_{total} \rangle$	total solute sieving coefficient calculated from the solute flux across the glomerular filtration surface, the solute flux across the four-layered barrier and the solute flux through the shunt at the junction between the two barrier
κ_{en}	Darcy permeability of the endothelial fenestrae
κ_{GBM}	Darcy permeability of GBM
κ_{mes}	Darcy permeability of the glomerular mesangium
μ	fluid shear viscosity
μ_{plasma}	plasma shear viscosity
τ	period of the shunt opening and closing
$\Phi_{en\text{-}Fluid}$	partition coefficient at the endothelial fenestrae-bulk fluid interface
$\Phi_{GBM\text{-}en}$	equilibrium partition coefficient at the GBM-endothelial fenestrae interface
$\Phi_{GBM\text{-}fluid}$	equilibrium partition coefficient at the GBM-bulk fluid interface
$\phi_{collagen}$	collagen volume fraction in GBM
ϕ_{GAG}	GAG volume fraction in GBM
$\phi_{GAG,en}$	GAG volume fraction in the endothelial fenestrae
ϕ_{total}	total volume fraction of fibers in GBM
C	local solute concentration
C_{shunt}	solute concentration in the shunt
D_∞	diffusivity of the unconfined solute
F	factor representing the increased hydrodynamic force exerted on the spherical particle in the fibrous medium scaled with Stokes' drag acting on the spherical particle in a bulk fluid
$\mathbf{F}_{shunt}$	hydrodynamic drag on a macromolecule passing through the shunt
f_P	dimensionless flow resistance of a slit channel consisting of two parallel plates
f_R	dimensionless flow resistance of a row of hindering cylinders
f_T	dimensionless flow resistance

$G(u)du$	fraction of the fluid volume flow rate through the interfiber spacing with the gap half-width being in the range of u and u+du
$g(u)du$	probability of the half-width of spacing between fibers at any position being between u and $u + du$
K_d^{en}	diffusive hindrance factor in the endothelial fenestrae
K_c^{en}	convective hindrance factor in the endothelial fenestrae
K_c^{GBM}	convective hindrance factor (the observable velocity of solutes during convection compared to the velocity of fluid at the same location in GBM)
K_d^{GBM}	diffusive hindrance factor (the ratio between the diffusivity of solutes in GBM and D_∞)
K_c^{mes}	convective hindrance factor of the solute in the mesangial matrix
K_d^{mes}	diffusive hindrance factor of the solute in the glomerular mesangium
k_{en}	hydraulic permeability of the endothelial cell layer
k_{ep}	hydraulic permeability of the epithelial cell layer
k_f	hydraulic permeability of the endothelial fenestrae
$k_{four\text{-}layered}$	hydraulic permeability of the four-layered barrier
k_{fs}	total hydraulic permeability of filtration surface
k_{GBM}	hydraulic permeability of GBM
k_{SD}	hydraulic permeability of the slit diaphragm
L_f	length of the endothelial fenestrae
L_{GBM}	GBM thickness
$L_{GBM\text{-}slit}$	distance between the slit diaphragm and the GBM downstream surface
$L_{mesangium}$	the thickness of glomerular mesangium
N	average number of shunts per glomerulus
$\mathbf{N}_{en}$	flux of solutes through the fenestrae of endothelium
$\mathbf{N}_{GBM}$	flux of solute flowing through the GBM
$\mathbf{N}_{mesangium}$	solute flux in the mesangium matrix
$\langle N_{shunt} \rangle$	axial component of the solute flux in the shunt
n_f	number of the fenestrae per ultrastructural subunit
Pe_{en}	the Peclet number representing the relative contribution between convection and diffusion on the transport of solute in the endothelial fenestrae
Pe_{GBM}^{bare}	Peclet number describing solute transport through the bare GBM
Pe_{GBM}^{intact}	Peclet number representing the relative contribution between convection and diffusion on solute in an intact GBM of which surfaces are partially obstructed by the endothelial cells and the epithelial foot processes
R_0	maximum radius of the shunt
$R(t)$	pore radius as a periodic function of time
$\langle R_{\mathrm{v}} \rangle$	average fluid flow rate transported through the shunt
$<R_0>$	mean value of R_0
r_c	radius of the fibers of the slit diaphragm
r_f	fiber radius
$r_f^{collagen}$	type-IV collagen radius

r_f^{GAG}	GAG radius
r_s	solute radius
S	factor from the steric interaction between particle and fiber
$S_{four\text{-}layered}$	surface area of the four-layered barrier
S_{fs}	surface area of glomerular filtration surface
$\mathbf{U}_{shunt}$	particle velocity in the shunt
u	half-width of the gap between adjacent fibers of the slit diaphragm
V_B	fluid velocity in Bowman's space
$\mathbf{v}_{en}$	fluid velocity in the endothelial fenestrae
$\mathbf{v}_{GBM}$	local fluid velocity in GBM
$\mathbf{v}_{mesangium}$	fluid velocity in the glomerular mesangium
v_{SD}	upstream fluid velocity far from the slit diaphragm
$\mathbf{v}_{shunt}$	fluid velocity in the shunt
v_{slit}	fluid velocity through a row of parallel cylinders with the gap half-width of the spacing between adjacent fibers
W	the width of an ultrastructural subunit
$W_{\text{4-layer}}$	the width of the four-layered barrier

References

Amsden B. Solute diffusion in hydrogels. an examination of the retardation effect. *Polym Gels and Networks* 6: 13-43, 1998. https://doi.org/10.1016/S0966-7822(97)00012-9

Andersen S, Blouch K, Bialek J, Deckert M, Parving H, Myers BD. Glomerular permselectivity in early stages of overt diabetic nephropathy. *Kidney Int* 58: 2129–2137, 2000. https://doi.org/10.1111/j.1523-1755.2000.00386.x.

Blouch K, Deen WM, Fauvel J, Bialek J, Derby G, Myers BD. Molecular configuration and glomerular size selectivity in healthy and nephrotic humans. *Am J Physiol* 273(3): F430-F437, 1997. https://doi.org/10.1152/ajprenal.1997.273.3.f430.

Boyd RF, Zydney AL. Sieving characteristics of multilayer ultrafiltration membranes. J Mem Sci 131(1-2): 155–165, 1997. https://doi.org/10.1016/S0376-7388(97)00045-8.

Brady JF. Hindered diffusion. American Institute of Chemical Engineers Annual Meeting, San Francisco, CA. Nov. 13-18, 320.1994.

Clague DS, Phillips RJ. Hindered diffusion of spherical macromolecules through dilute fibrous media. *Phys Fluids* 8: 1720 –1731, 1996. https://doi.org/10.1063/1.868884

Clague DS, Phillips RJ. A numerical calculation of the hydraulic permeability of three-dimensional disordered fibrous media. *Phys Fluids A* 9: 1562-1572, 1997. https://doi.org/10.1063/1.869278

Deen WM, Lazzara MJ, Myers BD. Structural determinants of glomerular permeability. *Am J Physiol Renal Physiol* 281: 579-596, 2001. https://doi.org/10.1152/ajprenal.2001.281.4.f579.

Drumond MC, Deen WM. Structural determinants of glomerular hydraulic permeability. *Am J Physiol* 266(1): F1-F12, 1994. https://doi.org/10.1152/ajprenal.1994b.266.1.f1.

Drumond MC, Deen WM. Hindered transport of macromolecules through a single row of cylinders: application to glomerular filtration. *ASME J Biomech Eng* 117(4): 414-422, 1995. https://doi.org/10.1115/1.2794202.

Edwards A, Daniels BS, Deen WM. Ultrastructural model for size selectivity in glomerular filtration. *Am J Physiol Renal Physiol* 276(6): F892-F902, 1999. https://doi.org/10.1152/ajprenal.1999.276.6.f892.

Ellis EN, Mauer SM, Sutherland DER, Steffes MW. Glomerular capillary morphology in normal humans. *Lab Invest* 60(2): 231-236, 1989.

Hunt SE, Dorfman KD, Segal Y, Barocas VH. A computational model of flow and species transport in the mesangium. *Am J Physiol Renal Physiol* 310: F222-F229, 2015. https://doi.org/10.1152/ajprenal.00182.2015

Johnson EM, Berk DA, Jain RK, Deen WM. Hindered diffusion in agarose gels: test of effective medium model. *Biophys J* 70: 1017-1026, 1996. https://doi.org/10.1016%2FS0006-3495(96)79645-5

Mattern KJ, Deen WM. "Mixing rules" for estimating the hydraulic permeability of fiber mixtures. *AIChE J* 54: 32-41, 2008. https://doi.org/10.1002/aic.11350

Ogston AG. The spaces in a uniform random suspension of fibers. *Trans Faraday Soc* 54: 1754-1757, 1958.

Oseen CW. Neure methoden und ergebnisse in der hydrodynamik. Akademische Verlagsgesellschaft, Leipzig. 1927.

Phillips RJ. A hydrodynamic model for hindered diffusion of proteins and micelles in hydrogels. *Biophys J* 79: 3350–3354, 2000. https://doi.org/10.1016%2FS0006-3495(00)76566-0

Punyaratabandhu N. Simulation of transport of spherical particles through hydrogel and row of parallel fibers for applications in glomerular filtration in normal and nephrotic humans. Chulalongkorn University, Bangkok. 2015.

Punyaratbandhu N, Kongoup P, Dechadilok P, Katavetin P, Triampo W. Transport of spherical particles through fibrous media and a row of parallel cylinders: applications to glomerular filtration. *ASME J Biomech Eng* 139: 121005, 2017. https://doi.org/10.1115/1.4037550.

Rice W, Hoek ANV, Paunescu TG, Huynh C, Goetze B, Singh B, Scipioni L, Stern LA, Brown D. High resolution helium ion scanning microscopy of the rat kidney. *PLoS One* 8(3): e57051, 2013. https://doi.org/10.1371/journal.pone.0057051.

Sangani AS, Acrivos A. Slow flow past periodic arrays of cylinders with application to heat transfer. *Int J Multiph Flow* 8(4): 343-360, 1982. https://doi.org/10.1016/0301-9322(82)90047-7

Sugihara-Seki M. Motion of a Sphere in a Cylindrical Tube Filled with a Brinkman Medium. Fluid Dyn Res 34(1): 59 - 76, 2004. doi: 10.1016/j.fluiddyn.2003.08.007

Tsay R, Weinbaum S. Viscous flow in a channel with periodic cross-bridging fibres: exact solutions and Brinkman approximation. *J Fluid Mech* 226: 125 – 148, 1991. https://doi.org/10.1017/S0022112091002318
